# Comparative genomic characterization of *Cellulosimicrobium funkei* isolate RVMD1 from Ma’an desert rock varnish challenges *Cellulosimicrobium* systematics

**DOI:** 10.3389/fmicb.2024.1445943

**Published:** 2024-11-07

**Authors:** Sulaiman M. Alnaimat, Saqr Abushattal, Saif M. Dmour

**Affiliations:** Department of Medical Analysis, Princess Aisha Bint Al-Hussein College of Nursing and Health Sciences, Al-Hussein Bin Talal University, Ma’an, Jordan

**Keywords:** Rock varnish, *Cellulosimicrobium funkei*, whole genome sequencing, phylogenomic, comparative genomic, taxonomic revision, Ma’an, Jordan

## Abstract

Desert environments harbor unique microbial communities. This study focuses on *Cellulosimicrobium funkei* isolate RVMD1, isolated from rock varnish in the Ma’an Desert. Initial identification was achieved using 16S rRNA gene sequencing, followed by whole-genome sequencing (WGS) for comprehensive characterization. The genome comprises 4,264,015 base pairs (857 contigs) with a high G + C content of 74.59%. A total of 4,449 proteins were predicted. Comparative analysis utilizing OrthoANI, ANI, AAI, and dDDH metrics suggests that RVMD1 belongs to the *C. cellulans* group, with the highest similarity to *C. funkei* (97.71% ANI). Phylogenomic analysis of 43 *Cellulosimicrobium* genomes revealed significant heterogeneity within the genus. Our results challenge current systematics, with *C. cellulans* potentially representing up to 9 distinct genomospecies. Isolate RVMD1 shows genetic adaptations to its desert environment, including genes for denitrification, oxygen and sulfur cycling, and diverse hydrogen metabolism. Pangenomic analysis uncovered a considerable number of unique genes within RVMD1, highlighting its genetic distinctiveness. Gene family expansions suggest evolution in response to stressors like UV radiation and nutrient limitation. This study represents the first whole-genome analysis of a bacterium isolated from Jordanian rock varnish, emphasizing the value of WGS in understanding microbial diversity and adaptation in extreme environments.

## 1 Introduction

The extreme habitats, such as arid and semi-arid desert regions, have become a focal point for researchers due to their unique microbial diversity (Shu and Huang, 2022). A prominent feature in arid and semi-arid regions is the formation of rock varnish, also referred to as desert varnish. This varnish forms a thin, dark layer on rocks, typically not exceeding a thickness of 200 μm. Its composition is quite complex and consists mainly of elements like oxygen, silicon, and aluminum (Alnaimat et al., 2017).

The exploration of the microbial and chemical characteristics of rock varnish and its formation has been significantly advanced by employing a variety of methods. Techniques such as shotgun metagenomics, elemental analysis, lipid analysis, biomarker analysis, and rock surface analysis have been crucial in investigating these properties (Lang-Yona et al., 2018; Ren et al., 2019). Recently, the discovery of the role of Cyanobacteria, particularly Chroococcidiopsis, in forming rock varnish and enhancing bacterial resilience, has opened new perspectives on the biogeochemical processes of Earth and Mars (Culotta and Wildeman, 2021).

The genus *Cellulosimicrobium* was initially introduced by Schumann and colleagues in 2001, with its first characterized and newly reclassified species, *C. cellulans* gen. nov., comb. nov., designated as the type species (Schumann et al., 2001). As of now, this genus has expanded to include seven validly published species, namely *C. composti*, *C. fucosivorans*, *C. funkei*, *C. marinum*, *C. protaetiae*, and *C. terreum*. Recently, *C. fucosivorans* was identified as a later heterotypic synonym of *C. composti* (Jiang and Han, 2023). As of September 26, 2023, there are 56 genome sequences from various members of the *Cellulosimicrobium* genus deposited in the NCBI database, accessible via.^1^ The majority are either *C. cellulans* or unspecified species within the *Cellulosimicrobium* genus.

This genus generally characterized as Gram-stain-positive, although they tend to decolorize quickly and they generate a substrate mycelium that eventually fragments into irregular, curved, and club-shaped rods, often arranged in a ‘V’ formation. As the medium becomes depleted, these rods transform into shorter rods or even spherical cells. These bacteria are non-spore-forming, catalase-positive, and display varying degrees of nitrate reduction. There are differences in motility among species in this genus. For example, *C. funkei* cells have one to five polar and/or lateral flagella and are motile, while *C. cellulans* are nonmotile (Schumann et al., 2001; Schumann and Stackebrandt, 2015). *Cellulosimicrobium* species have been isolated from soil (Yoon et al., 2007), clinical samples (Brown et al., 2006) and from marine sponges (Li, 2009; Antony et al., 2009). Despite being considered a rare pathogen, *Cellulosimicrobium* spp. may cause life-threatening conditions, such as endocarditis, particularly in immunocompromised individuals and those carrying foreign objects (Monticelli et al., 2019; Rivero et al., 2019). A specific case demonstrated *C. cellulans* being isolated from a patient who developed acute renal failure after a prolonged ICU stay for sepsis encephalopathy. This evidence supports its potential as an opportunistic pathogen in critically ill patients (Delport et al., 2014).

The use of whole-genome sequencing (WGS) for the comprehensive characterization of bacterial genetic diversity has become a standard approach, enabling a deeper understanding of their genomic structures and functional attributes. As part of our ongoing research focusing on microbial diversity, particularly the diversity of actinobacteria in desert varnishes, the purpose of this study is to determine the taxonomic status and comprehensively describe the phylogenetic, genomic, and taxonomic characteristics of *C. funkei* isolate RVMD1, isolated from rock varnish in the Ma’an Desert, Jordan. This is achieved by employing a multiphase classification approach that integrates whole-genome shotgun sequencing with rRNA gene amplicon analyses. To the best of our knowledge, this study represents the first instance of whole-genome sequencing for bacteria isolated from rock varnish in Jordan.

## 2 Materials and methods

### 2.1 Site description and sample collection

Several samples of desert rock varnish were aseptically collected from a semi-arid region close to Ma’an city, Jordan, at coordinates 30.188836, 35.639121. Approximately 50 mm of rain falls annually in this region. Aseptically selected rocks with relatively flat surfaces were immediately sealed in sterile aluminum foil for transportation. Once the rock specimens were transported to the laboratory, they were placed in a laminar flow bench, where a flame-sterilized coarse bit was used to grind them into varnish powder. The powder was then stored at −4°C. Bacteria cultivation was performed within 24 h of sample collection.

### 2.2 Bacteria isolation and enumeration

A 0.1 g sample of powdered rock varnish was placed directly onto Luedemann medium (DSMZ medium 877), supplemented with 50 μg/mL of Cycloheximide to prevent fungal growth. Following a 72-h incubation period at 37°C, colonies exhibiting distinct shapes and colors were identified, isolated, and then streak purified for further analysis.

### 2.3 Identification of isolate through 16S rRNA gene amplification and sequencing

The isolated samples underwent DNA extraction using the G-spin Total DNA Extraction Mini Kit (iNtRON Biotechnology, Suwon, Korea). Resultant DNA then served as a template for 16S rDNA amplification via PCR. The extraction process was carried out in line with the manufacturer’s guidelines. The SSU rRNA gene was amplified using the bacterial forward primer 27F (5′-AGRGTTYGATYMTGGCTCAG-3′) paired with the 1492R primer (5′-RGYTACCTTGTTACGACTT-3′). PCR product was purified using the PCR quick-spin PCR Product Purification Kit as per the manufacturer’s instructions, and sequenced by MACROGEN (Korea) using the Sanger method.

The obtained 16S rRNA gene sequences were analyzed and cross-referenced with the EzTaxon database^2^ and EzBioCloud Pro 16S-ID app (Yoon et al., 2017). A partial sequence of the 16S ribosomal RNA (rRNA) gene, encompassing 1,251 base pairs (bp), was obtained from the isolate RVMD1. This sequence has been successfully deposited in the NCBI GenBank database and is accessible under the accession number OR570906. 16S rRNA gene-based phylogenetic tree was constructed using Protologger.^3^ Sequences were aligned using MUSCLE (v3.8.31) with default settings, and further processed using FastTree (v2.1.7) with the GTR model to generate the phylogenetic tree. Taxonomic assignment involved identifying the closest relatives within the SILVA Living Tree Project based on sequence identity. Only species with validly published names from the DSMZ nomenclature list were included to ensure accurate classification (Hitch et al., 2021). The phylogenetic tree was visualized using the Interactive Tree Of Life (ITOL) online tool^4^ (Letunic and Bork, 2021) and subsequently edited in Inkscape (version 1.0).

### 2.4 Whole genome sequencing, assembly, annotation, and analysis of genomic features

As outlined in the protocol for Gram-positive bacteria, genomic DNA was extracted using the G-spin Total DNA Extraction Mini Kit (iNtRON Biotechnology, Suwon, Korea). The purity and integrity of obtained DNA were assessed using the Nabi-UV/Vis Nano Spectrophotometer from MicroDigital, South Korea.

The genome of isolate RVMD1 was sequenced using Illumina NextSeq 2000 (PE 150 bp, 15 M reads/sample) at EzBiome Inc., (Gaithersburg, MD, USA). Quality control of raw reads was checked with MultiQC, v.1.11 (Ewels et al., 2016). The reads were assembled *de novo* using SPAdes v.3.13.0 (Prjibelski et al., 2020). CheckM v1.0.18 (Parks et al., 2015) and QUAST v4.4 (Gurevich et al., 2013) through^5^ (Arkin et al., 2018) were used to assess genome quality.

The genome sequence was fully annotated using NCBI Prokaryotic Genome Annotation Pipeline (PGAP) (Tatusova et al., 2016) and Bacterial Bioinformatics Resource Center (BV-BRC)^6^ (Olson et al., 2023), which provided a comprehensive analysis of its genetic makeup. Additional insights into various genomic features were obtained through the use of other tools, including the Microbial Genomes Atlas (MiGA) webserver^7^ (Rodriguez-R et al., 2018), the GTDB-Tk in the KBase platform (Chaumeil et al., 2019), EzBiome Genome-ID^8^ and Galaxy Protologger (see text footnote 3) (Hitch et al., 2021). Circular genome map was generated through Proksee web-based tool^9^ (Grant et al., 2023). The analysis of gene clusters and loci associated with antibiotic resistance and iron acquisition systems were retrieved using the SEED viewer of the RAST platform^10^ (Aziz et al., 2008; Overbeek et al., 2014).

### 2.5 Whole genome-based taxonomic placement, relatedness indices, and comparative phylogenomic analysis

The initial taxonomic status of isolate RVMD1 was identified by the whole genome-based bacterial identification service provided by EzBiome Genome-ID (see text footnote 8) (Ha et al., 2019). This service uses average nucleotide identity (ANI) to compare similarity values with a reference database during its identification process.

After initially identified through 16S rRNA and whole-genome sequencing, the taxonomic status of *C. funkei* RVMD1 was further clarified by a comprehensive phylogenomic analysis of 43 genomes from its genus in RefSeq as of September 2023, excluding atypical.^11^ This analysis encompassed a multifaceted approach, including genome-scale GBDP tree analysis via TYGS^12^ (Meier-Kolthoff et al., 2022), the analysis also involved calculating pairwise average nucleotide identity (ANI) using BLAST (ANIb) through JSpeciesWS^13^ (Richter et al., 2016). Digital DNA–DNA hybridization (dDDH) percentages were determined *in silico* using the Genome-to-Genome Distance Calculator (dGGDC 3.0) formula *d4* (a.k.a. GGDC formula 2) (Meier-Kolthoff et al., 2022) applying a 70% species delineation threshold (Chun et al., 2018), leveraging the BLAST method as per the formula available at.^14^ Additionally, ANI/AAI matrix values for our isolate, *C. funkei* RVMD1, along with 43 other genomes, were calculated using the enveomics collection (Rodriguez-R and Konstantinidis, 2016).^15^ Matrix data were represented as a heatmap, generated using TBtools-I (Chen et al., 2023). The AAI-profiler server (Medlar et al., 2018)^16^ was employed for visualizing scatter plots and taxonomic profiles, confirming taxonomic identities, facilitating proteome-wide sequence searches, and validating isolate classification based on AAI-profile analysis, by computing AAI between a query proteome and all target species in the UniProt database.

In-depth comparative genome analyses were undertaken *for C. funkei* RVMD1 in relation to six reference species of *Cellulosimicrobium* genus (*C. protaetiae* BI34, *C. cellulans* ORNL-0100, *C. funkei* JCM 14302, *C. marinum* NBRC 110994, *C. composti* SE3 and *C. arenosum* KCTC 49039), genome sequences were retrieved from the National Center for Biotechnology Information (NCBI) up to September 2023. The analysis involved constructing a phylogenetic tree using OrthoANI values computed with the Orthologous Average Nucleotide Identity Tool (OAT)(Lee et al., 2016) on the EzBioCloud platform.^17^ Heatmap techniques were applied for visualizing OrthoANI to identify nucleotide similarities between RVMD1 and reference species. Circos chord diagram (Krzywinski et al., 2009) was employed to display OGRI metrics (AAI, ANI, dDDH) for an in-depth comparative view.^18^ Detailed visual fast whole-genome similarity analysis was performed using the FastANI 1.3.3 tool (Jain et al., 2018) available on the Proksee website (Grant et al., 2023). Genes associated with siderophore biosynthesis, heavy metal resistance, virulence, and cold and heat shock responses were analyzed using the BV-BRC platform (Olson et al., 2023). The results are illustrated in a horizontal stacked bar chart created using Google Sheets. The distribution of MicroTrait bioelement family genes was analyzed using HMMs of MicroTrait Bioelement families (v1.9.1) available on KBase (Karaoz and Brodie, 2022), the heatmap was created using TBtools-I (Chen et al., 2023). Pangenomic comparisons were executed using Build Pangenome with OrthoMCL - v2.0 in the Kbase platform (Arkin et al., 2018) and with IPGA, web server available at,^19^ configured with its default settings (Liu et al., 2022). The contraction and expansion of gene families in *C. funkei* RVMD1 and reference genomes were analyzed using CAFE5, with the process facilitated through OrthoVenn3 (Sun et al., 2023), accessible at.^20^

## 3 Results and discussion

### 3.1 Isolation and 16S rRNA gene sequencing based characterization

In our ongoing exploration of microbial diversity, particularly focusing on the diversity of actinobacteria present in extreme habitats, a bacterium designated RVMD1 was isolated from a desert rock varnish sample. A characterization based on 16S rRNA gene sequencing was initially started. The obtained partial 16S rRNA gene sequence (1,251 bp) of isolate RVMD1, submitted to GenBank with the accession number OR570906, has been analyzed and summarized in Table 1, using the EzBioCloud Pro 16S-ID app (Yoon et al., 2017). This table ranks 10 species based on their sequence similarity to RVMD1, highlighting both the species name and their respective genome group. At the top of the list is *C. cellulans* from the *C. cellulans* group, showing the highest similarity to RVMD1 with 99.84% identification. It is followed by *C. funkei*, also of the same group, with a 99.68% match. Both species are well-known for their genetic similarity (Brown et al., 2006). Other species like *C. marinum*, *C. fucosivorans* (from the *C. composti* group), and *C. composti* (same group) also display high similarities, ranging from 99.52 to 99.36%. Notably, *C. fucosivorans*, originally listed in the *C. composti* group with a 99.44% similarity, was recently identified as a later heterotypic synonym of *C. composti*, according to Jiang and Han (2023). The list also includes species without specified genome groups like *C. protaetiae*, *C. terreum*, *Krasilnikoviella muralis*, *C. arenosum*, and *K. flava*, with similarities spanning from 99.2 to 97.45%. The alignment of the top 16S rRNA gene sequencing matches for RVMD1 is neatly outlined in Supplementary Figure S1. An intricate analysis identified taxa closely related to RVMD1 isolate detailed in Supplementary Table S1, used genes extracted from the whole genome, including 16S rRNA, *recA*, *rplC*, and Mash identity. The 16S rRNA gene sequences of isolate RVMD1 were compared between Sanger sequencing (1,251 bp) and whole-genome sequencing (WGS, 1,467 bp). Two minor nucleotide differences were identified (T ↔ C and C ↔ T), as detailed in Supplementary Figure S2.

**Table 1:** A summary of top hits from 16S rRNA gene sequencing analysis of bacterial isolate RVMD1 in EzBioCloud Pro 16S-ID app.

Rank	Species name	Genome group	Identification (%)	Query coverage (%)	Reference coverage (%)
1	*C. cellulans*	*C. cellulans* group	99.84	100	86.6
2	*C. funkei*	*C. cellulans* group	99.68	100	86.6
3	*C. marinum*	–	99.52	100	86.6
4	*C. fucosivorans*	*C. composti* group	99.44	100	86.6
5	*C. composti*	*C. composti* group	99.36	100	86.6
6	*C. protaetiae*	–	99.20	100	86.6
7	*C. terreum*	–	97.77	100	86.6
8	*Krasilnikoviella muralis*	–	97.60	100	86.6
9	*C. arenosum*	–	97.53	100	86.6
10	*Krasilnikoviella flava*	–	97.45	100	86.7

**Figure 1:**
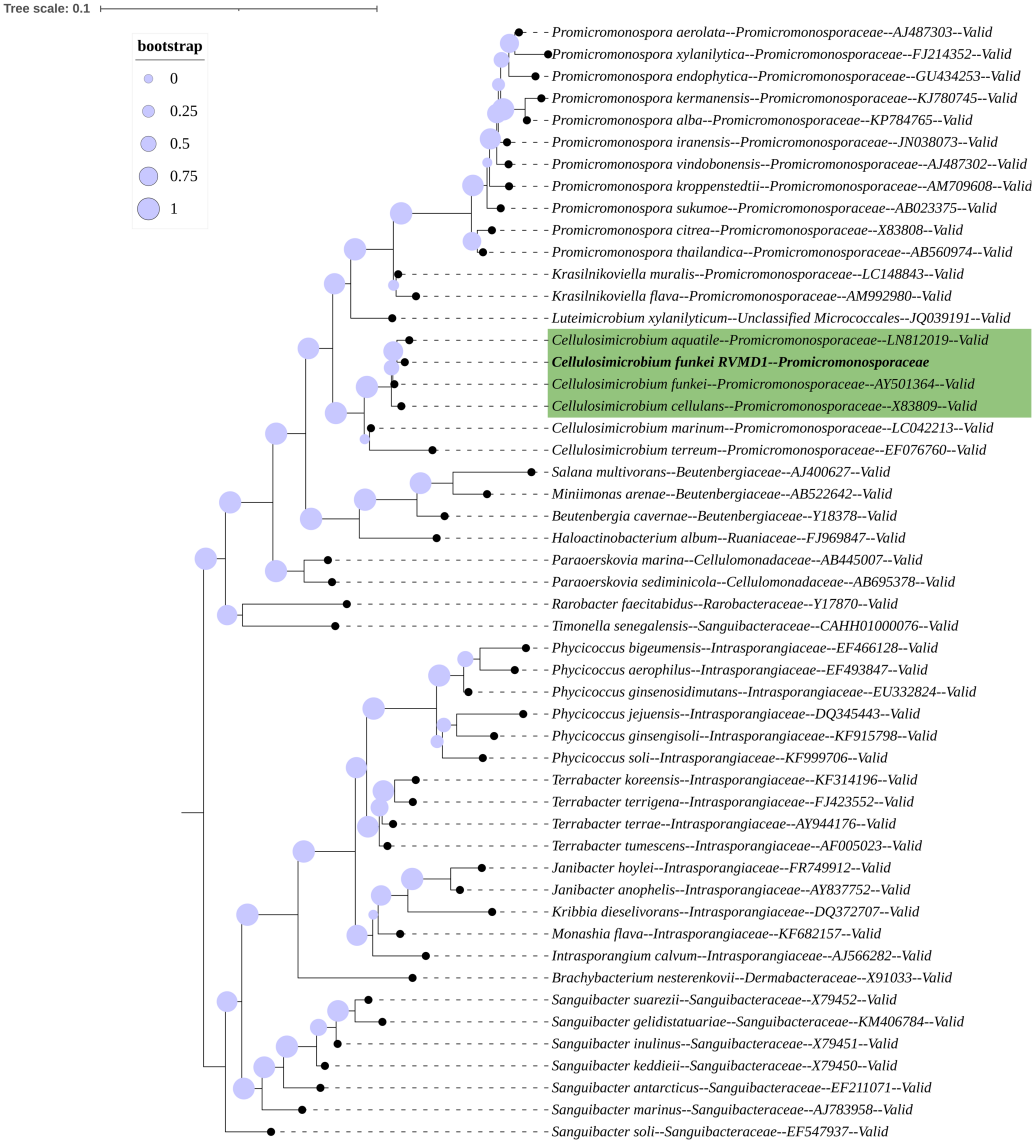
16S rRNA gene-based mid-point rooted maximum-likelihood phylogenetic tree of *C. funkei* RVMD1, constructed using Protologger, highlights closest relatives from the SILVA Living Tree Project. The tree was modified using the Interactive Tree Of Life (iTOL) v5. Proportional circles on the branches represent bootstrap values. Species belonging to the *Sanguibacteraceae* family serve as the outgroup.

The 16S rRNA gene-based mid-point rooted maximum-likelihood phylogenetic tree for isolate RVMD1, constructed with Protologger and referenced against the SILVA Living Tree Project, is illustrated (Figure 1). The tree confirms that RVMD1 belongs to the *C. cellulans* group, with *C. aquatile* shown as the closest relative, followed by *C. funkei* and *C. cellulans*. However, according to a recent nomenclatural revision, *C. aquatile*, as identified by Sultanpuram et al. (2015), is now considered a synonym of *C. funkei* (Nouioui et al., 2018). This means that what was previously thought to be separate species, *C. aquatile* and *C. funkei*, are now recognized as the same species, which is reflected in the close clustering of these names in the phylogenetic tree.

### 3.2 Genomic features and annotations

**Figure 2:**
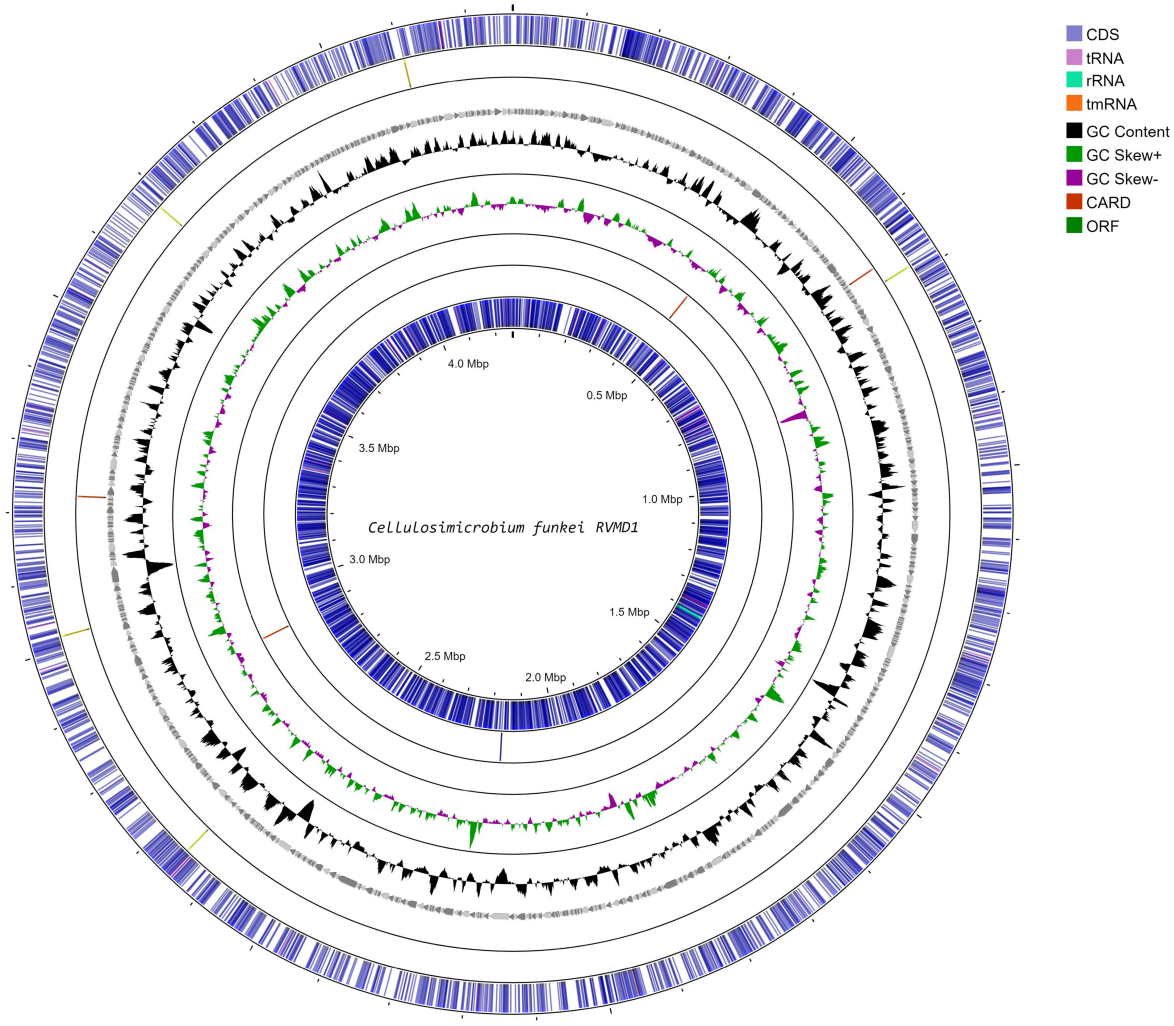
Circular genome map of *C. funkei* isolate RVMD1 generated through Proksee web-based tool. The tracks display coding sequences on forward and reverse strands (CDS), tRNAs, rRNAs, GC content, and GC skew (black/gray graph), with antibiotic resistance genes (CARD) and open reading frames (ORF) interspersed, illustrating the detailed genomic structure.

The integration of data from bioinformatics resources such as MiGA, BV-BRC, NCBI PGAP, GTDB-Tk on KBase, and Galaxy Protologger has yielded a comprehensive overview of the genomic features of the *C. funkei* RVMD1, as shown in Table 2 and Figure 2. The genome is comprised of 857 contigs with a total length of 4,264,015 base pairs, with an N50 value of 9,300 bp. The largest contig identified measured 36,920 bp nucleotides. Factors such as the starting material, the sequencing technique used, and the quality of the produced sequences impacted the number and lengths of the contigs generated (Schwengers et al., 2020). The G + C content of 74.59% is particularly high, however, the values of genome size and G + C contents fall within the range observed for all recognized strains within the genus *Cellulosimicrobium*, as documented in Supplementary Table S4. Quality metrics, including completeness (98.55%) and contamination (0.87%), along with consistency scores, coarse consistency of 98.4 and fine consistency of 94.3, suggest a high-quality genome assembly (Supplementary Figures S3, S4). The quality of the genome data exceeds the minimum standards for taxonomic applications, as outlined by Chun et al. (2018) and Riesco and Trujillo (2024). Gene prediction analysis indicates a substantial proteome, with 4,449 proteins predicted, each averaging 288.3805 amino acids in length. This count of proteins is the highest ever recorded among all submitted genomes within this genus (Supplementary Table S4). It is important to note that this elevated protein count may be influenced by assembly inaccuracies, possibly due to the high number of contigs. The coding density stands at 90.27%, suggesting an efficient genomic coding capability. Isolated from a harsh desert environment, the identification of 103 out of 106 essential genes in *C. funkei* RVMD1, with duplications in some, could underlines its evolutionary adaptations for resilience and survival (Rosconi et al., 2022). Functional analysis uncovered a considerable number of transporters (144), secretion genes (14), and unique enzymes (643).

**Table 2:** Comprehensive genomic features of *C. funkei* isolate RVMD1.

Category	Feature	Value	Source(s)
Genome Assembly	Contigs	857	MiGA, BV-BRC, NCBI PGAP
	Total length	4,264,015 bp	MiGA, BV-BRC, NCBI PGAP
	N50	9,300 bp	MiGA, BV-BRC
	Longest sequence	36,920 bp	MiGA
	G + C content	74.59%	MiGA, BV-BRC, GTDB Tk-KBase, NCBI PGAP
	G-C skew	0.14%	MiGA
	A-T skew	0.15%	MiGA
Genome Quality	Completeness	98.55%	CheckM –Kbase
	Contamination	0.87%	CheckM –Kbase
	Coarse Consistency	98.4	BV-BRC
	Fine Consistency	94.3	BV-BRC
	Genome Quality Assessment	Good	BV-BRC
Gene Prediction	Predicted proteins	4,449	MiGA
	Average protein length	288.3805 aa	MiGA
	Coding density	90.27%	MiGA
	CDS	4,511	BV-BRC
	tRNA	52	BV-BRC, MiGA
	rRNA	3	BV-BRC
	Genes (total)	4,350	NCBI PGAP
	Genes (coding)	4,232	NCBI PGAP
	Genes (RNA)	57	NCBI PGAP
	Pseudo Genes (total)	61	NCBI PGAP
Essential Genes	Essential genes found	103/106	MiGA
	Multiple Copies	*L28*, *Era*, *L32* (2 each)	MiGA
	Missing Genes	*glyQ*, *valS*, *lysidine_TilS_N*	MiGA
	UBCG^*^ Recovery	92.39% (85/92)	EzBiome Genome-ID
Functional Analysis	Number of transporters	144	GTDB Tk-KBase, Protologger
	Number of secretion genes	14	GTDB Tk-KBase, Protologger
	Number of unique enzymes	643	GTDB Tk-KBase, Protologger

Data were compiled from multiple sources: The Microbial Genomes Atlas (MiGA) webserver, Bacterial Bioinformatics Resource Center (BV-BRC) PATRIC, NCBI Prokaryotic Genome Annotation Pipeline (PGAP), functional analysis via the GTDB-Tk in the KBase platform and Galaxy Protologger.

* UBCG, Universal Bacterial Core Gene.

**Figure 3:**
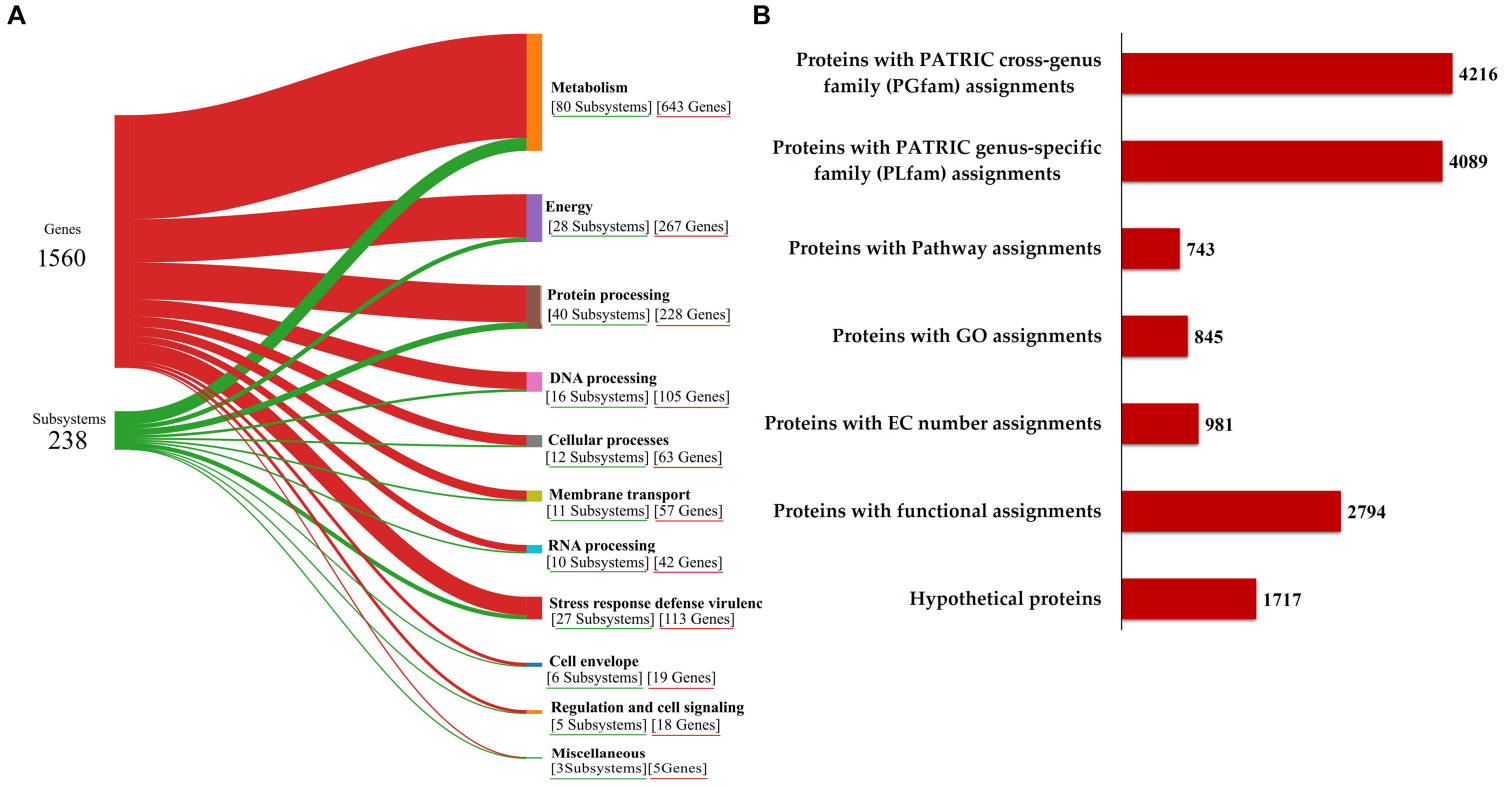
Functional characterization of the *C. funkei* RVMD1 genome. **(A)** Sankey diagram illustrating the distribution of genes into subsystems classified by biological functions. **(B)** Summary of protein annotations detailing cross-genus and genus-specific family assignments, pathway connections, GO categorizations, EC number designations, and other functional annotations, along with hypothetical proteins, all as annotated by BV-BRC.

The functional annotation of the genome, illustrated in Sankey diagram (Figure 3A), outlines the allocation of 1,560 genes into 238 subsystems, with a pronounced emphasis on metabolic functions (643 genes across 80 subsystems), energy (267 genes in 28 subsystems), protein processing (228 genes in 40 subsystems), DNA processing (105 genes in 16 subsystems), and notably, stress response defense virulence (113 genes in 27 subsystems), supporting the RVMD1 isolate adaptation to its extreme habitat. The annotation revealing 1,717 hypothetical proteins and 2,794 with functional assignments underscores the vast unknown in microbial genetics and the potential for groundbreaking discoveries (Figure 3B). This insight highlights the challenge of functionally uncharacterized genes in microbial systems and points to significant avenues for future exploratory research (Wirbel et al., 2024). Among the proteins with functional assignments were 981 with Enzyme Commission (EC) numbers (Schomburg et al., 2004), 845 with Gene Ontology (GO) assignments (Ashburner et al., 2000), and 743 mapped to KEGG pathways (Kanehisa et al., 2016). Additionally, the PATRIC annotation revealed 4,089 proteins belonging to genus-specific protein families (PLFams) and 4,216 proteins associated with cross-genus protein families (PGFams) (Davis et al., 2016).

**Figure 4:**
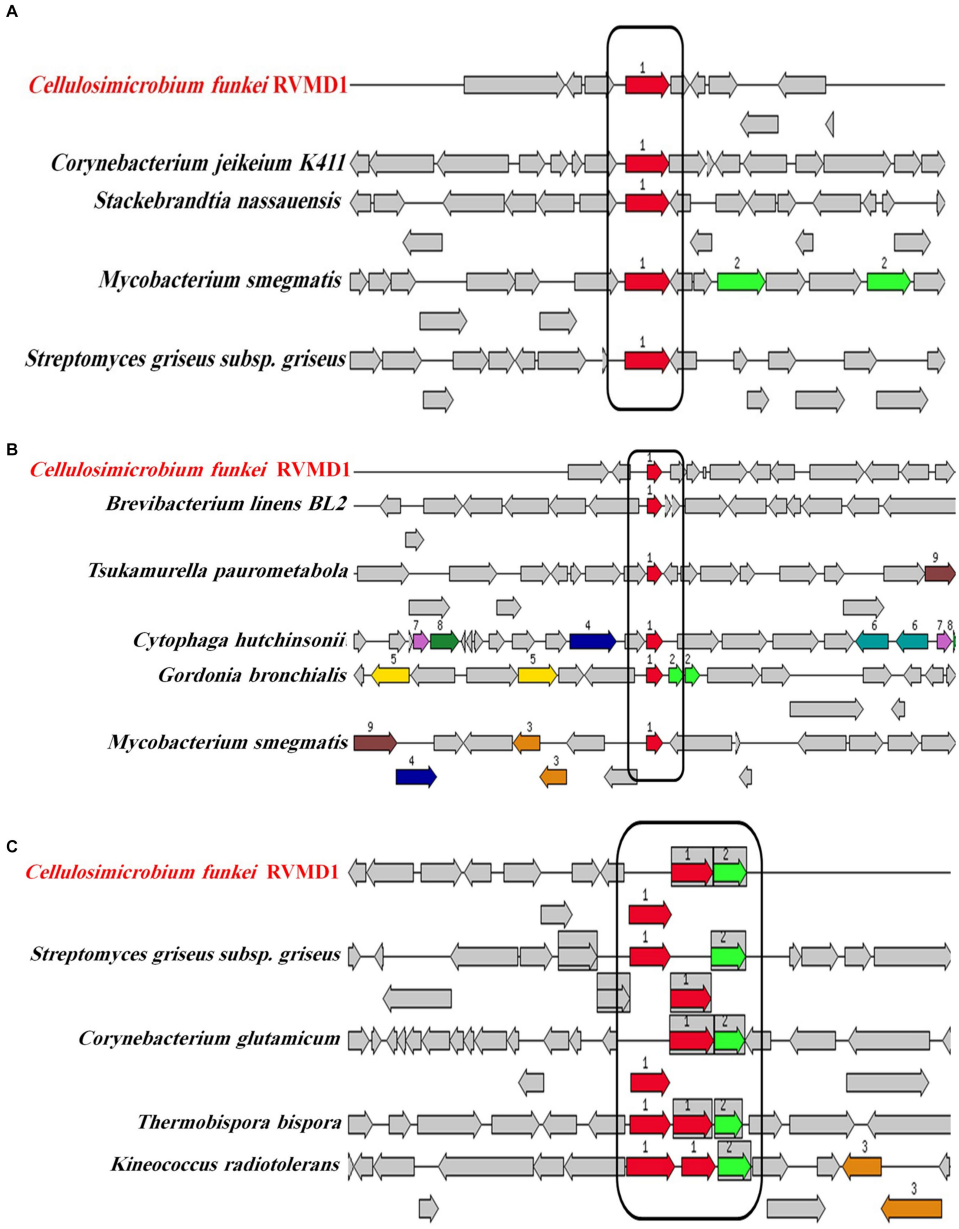
Gene clusters and loci associated with antibiotic resistance and iron acquisition systems in *C. funkei* RVMD1 in comparison to other bacteria, as characterized by the RAST SEED viewer. The graphic is centered on the focus gene, which is red and numbered (1). **(A)** Presents chloramphenicol resistance genes. **(B)** Displays rifampin resistance genes. **(C)** Depicts siderophore transport system genes with ATPase components (red) and permease components (green). Conserved regions across species are boxed.

Encouraged by reports that species such as *C. cellulans* and *C. funkei* within the genus *Cellulosimicrobium* have been documented as pathogenic to humans, with 15 cases of *Cellulosimicrobium* sp. bacteremia reported (Rivero et al., 2019; Le Ho et al., 2024), virulence factors were investigated in the RVMD1 genome. The results are shown in Figure 4A, which illustrates chloramphenicol resistance gene clusters demonstrating conservation across species including *Corynebacterium jeikeium* and *Streptomyces griseus*. Figure 4B presents rifampin resistance genes, with colored blocks indicating specific gene functions, involving a variety of bacterial species. Figure 4C highlights siderophore transport system gene clusters, featuring ATPase and permease components, across species such as *Corynebacterium glutamicum* and *Kineococcus radiotolerans*. Table 3 details the aforementioned antibiotic resistance determinants, including a chloramphenicol resistance gene (*cml*) with accession WP_005297378.1, located on contig 241 at coordinates 4,408–5,592 in the forward direction, displaying 63.78% identity and 99.74% reference coverage. Similarly, a rifamycin resistance gene (*arr*) with accession WP_063857695.1 was identified on contig 77, from 9,294 to 9,704, also in the forward direction, with 51.16% identity and 84% reference coverage. The presence of full-length genes associated with antimicrobial resistance (AMR) in a genome should not be regarded as conclusive evidence of a resistant phenotype (Elarabi et al., 2023). Supplementary Tables S2, S3 outline in detail the potential virulence and antimicrobial resistance genes in the studied genome. Supplementary Table S2 classifies virulence factor genes identified through TrueBac™ and VFDB, showing gene similarity to known sequences, while Supplementary Table S3 details AMR genes detected using the Genome Annotation Service in PATRIC (BV-BRC) with a *k*-mer-based method. While the findings were sourced from TrueBac™ and (BV-BRC), independent validation of these results was not possible by our team.

**Table 3:** Antibiotic resistance determinants in bacterial isolate RVMD1 as identified by whole genome analysis using EzBiome Genome-ID.

Class	Subclass	Gene	Form of AMR	Accession	Contig	Location	Dir.	Iden. (%)	Ref. cov. (%)
Phenicol	Chloramphenicol	*cml*	Gene	WP_005297378.1	241	4,408…5,592	→	63.78	99.74
Rifamycin	Rifamycin	*arr*	Gene	WP_063857695.1	77	9,294…9,704	→	51.16	84.00

### 3.3 Whole genome-based taxonomic placement

Building upon the initial findings that suggested the RVMD1 genome belonged to the genus *Cellulosimicrobium* based on the 16S rRNA gene sequence, a more comprehensive investigation was undertaken through whole-genome analysis. This was due to increasing evidence which suggests that the 16S rRNA gene sequence offers limited resolution in distinguishing between closely related species within certain genera (Nouioui et al., 2018; Wu et al., 2020; Rahi et al., 2024). This subsequent analysis employed EzBiome Genome-ID (Ha et al., 2019), a web-based commercial service, to leverage Average Nucleotide Identity (ANI) for in-depth comparison with a reference database. This approach aimed to definitively elucidate and confirm the accurate taxonomic position of isolate RVMD1. As shown in Table 4, this crucial step identified *C. funkei* and *C. cellulans*, both members of the *C. cellulans* group, as the closest relatives. The ANI results revealed *C. funkei* as the top match, exhibiting a nucleotide identity of 97.71%, along with query and reference coverage of 66.43 and 58.20%, respectively. *C. cellulans* closely followed, with an identity of 95.70%, and query and reference coverage values of 68.82 and 58.95%, respectively. These findings, surpassing the species boundary threshold of 94.0–96.0% (Chun et al., 2018), indicate a significant genetic closeness and suggest that the organisms belong to the same species (Aviles and Kyndt, 2021). While the remaining top hits (*C. protaetiae, C. fucosivorans, C. composti, C. marinum, C. terreum, C. arenosum, Isoptericola jiangsuensis* and *I. dokdonensis*) scored ANI values from 88.99 to 79.48, which are below the threshold boundary for species delineation. This confirms that our isolate is a species from *C. cellulans* group and is closely related to the funkei species; therefore, we designated it as *C. funkei* RVMD1. These results also support the latest reclassification that *C. fucosivorans* and *C. composti* are the same species (Jiang and Han, 2023).

**Table 4:** Top average nucleotide identity (ANI) hits for bacterial isolate RVMD1 whole genome: a comparative analysis of closely related species using EzBiome Genome-ID.

Rank	Species	Genome group	Iden. (%)	Query cov. (%)	Ref. cov. (%)
1	*C. funkei*	*C. cellulans* group	97.71	66.43	58.20
2	*C. cellulans*	*C. cellulans* group	95.70	68.82	58.95
3	*C. protaetiae*	–	88.99	53.7	43.11
4	*C. fucosivorans*	*C. composti* group	88.87	53.07	48.03
5	*C. composti*	*C. composti* group	88.72	53.22	48.01
6	*C. marinum*	–	84.61	39.52	39.55
7	*C. terreum*	–	83.79	40.49	34.62
8	*C. arenosum*	–	83.29	35.53	34.57
9	*Isoptericola jiangsuensis*	–	79.71	23.00	21.73
10	*Isoptericola dokdonensis*	–	79.48	22.65	22.63

**Figure 5:**
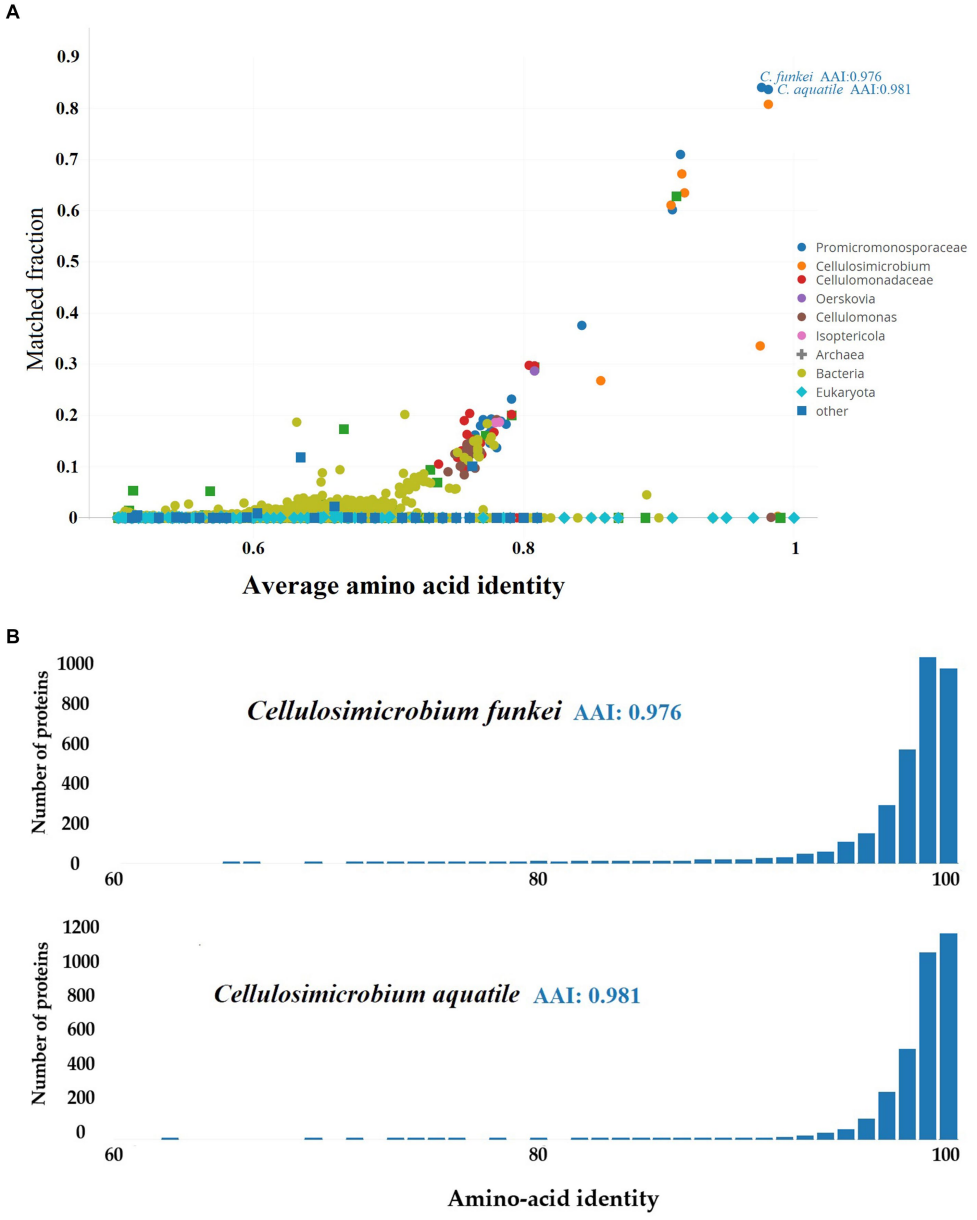
Proteomic analysis of *C. funkei* RVMD1 using AAI-Profiler. **(A)** AAI-Profiler scatterplot analysis of *C. funkei* RVMD1 proteome including distribution of sequence identity matches for proteins against the UniProt database. **(B)** AAI histograms of proteome similarity between *C. funkei* RVMD1 and its closest UniProt matches, *C. funkei* and *C. aquatile.*

On the other hand, the taxonomic resolution analysis employing Average Amino-acid Identity (AAI) (Figure 5), conducted on *C. funkei* RVMD1 against the UniProt database using AAI-Profiler, also revealed a high proteomic similarity between RVMD1 and both *C. funkei* and *C. aquatile*, with respective similarities of 97.6 and 98.1%. These values exceed the species delineation threshold, further supporting the conclusion drawn from the aforementioned ANI analysis that our isolate is *C. funkei*. Notably, *C. aquatile* is no longer considered a separate species; it is now recognized as a synonym of *C. funkei*, as described by Nouioui et al. (2018). The AAI-Profiler web server was used to generate a similarity matrix heatmap for essential genes extracted by the Microbial Genomes Atlas (MiGA) from the *C. funkei* RVMD1 genome, comparing them against the UniProt database, which substantiated that the top related species is, as previously concluded, *C. funkei* (Supplementary Figure S5).

### 3.4 Genomic insights challenge current systematics within the *Cellulosimicrobium* genus

Our investigation into the taxonomic position of our isolate revealed significant uncertainties surrounding the classification of existing genomes within the *Cellulosimicrobium* genus, particularly in light of recent whole-genome sequencing data (Nouioui et al., 2018). This observation aligns well with the many reclassifications documented in recent literature (Nouioui et al., 2018; Jiang and Han, 2023). It also supports the observation that this genus exhibited significant heterogeneity, with only a few well-defined species (Aviles and Kyndt, 2021), highlighting the limitations of the current framework. As shown in Supplementary Table S4, around 42% (18/43) of the analyzed genomes are assigned as *C. cellulans* species, while a significant portion (33%) remains unclassified.

In response, initiation of a thorough phylogenomic study was deemed necessary to gain clearer insights into the taxonomic place of the isolate within the genus hierarchy, specifically targeting the resolution of unclear species boundaries across the genus. The employed approach, incorporating the latest genomic data, has the potential to yield a more accurate and robust taxonomic distribution with refined positions for all genomes in *Cellulosimicrobium* genus. The analysis focused on 43 genomes retrieved from the *Cellulosimicrobium* genus within NCBI RefSeq as of September 2023 (excluding atypical genomes). To address the observed classification uncertainties, we employed a multifaceted approach encompassing GBDP (Genome BLAST Distance Phylogeny) tree analysis at the genome scale through the Type (Strain) Genome Server (TYGS) (Meier-Kolthoff et al., 2013; Meier-Kolthoff et al., 2022), alongside Genome-to-Genome Distance Calculator (GGDC) analysis, implementing a 70% species delineation threshold. Additionally, the study included the computation of pairwise average nucleotide identity (ANI) using BLAST (ANIb) via JSpeciesWS, and the determination of ANI/AAI (Average Amino Acid Identity) matrix values through the enveomics collection (Rodriguez-R and Konstantinidis, 2016).

**Figure 6:**
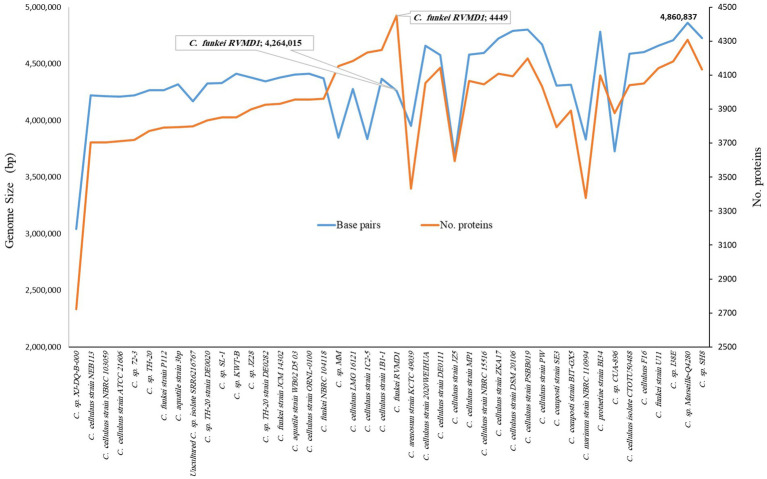
Comparison of genome size and protein counts in 43 *Cellulosimicrobium* genomes and *C. funkei* RVMD1 based on TYGS analysis.

**Figure 7:**
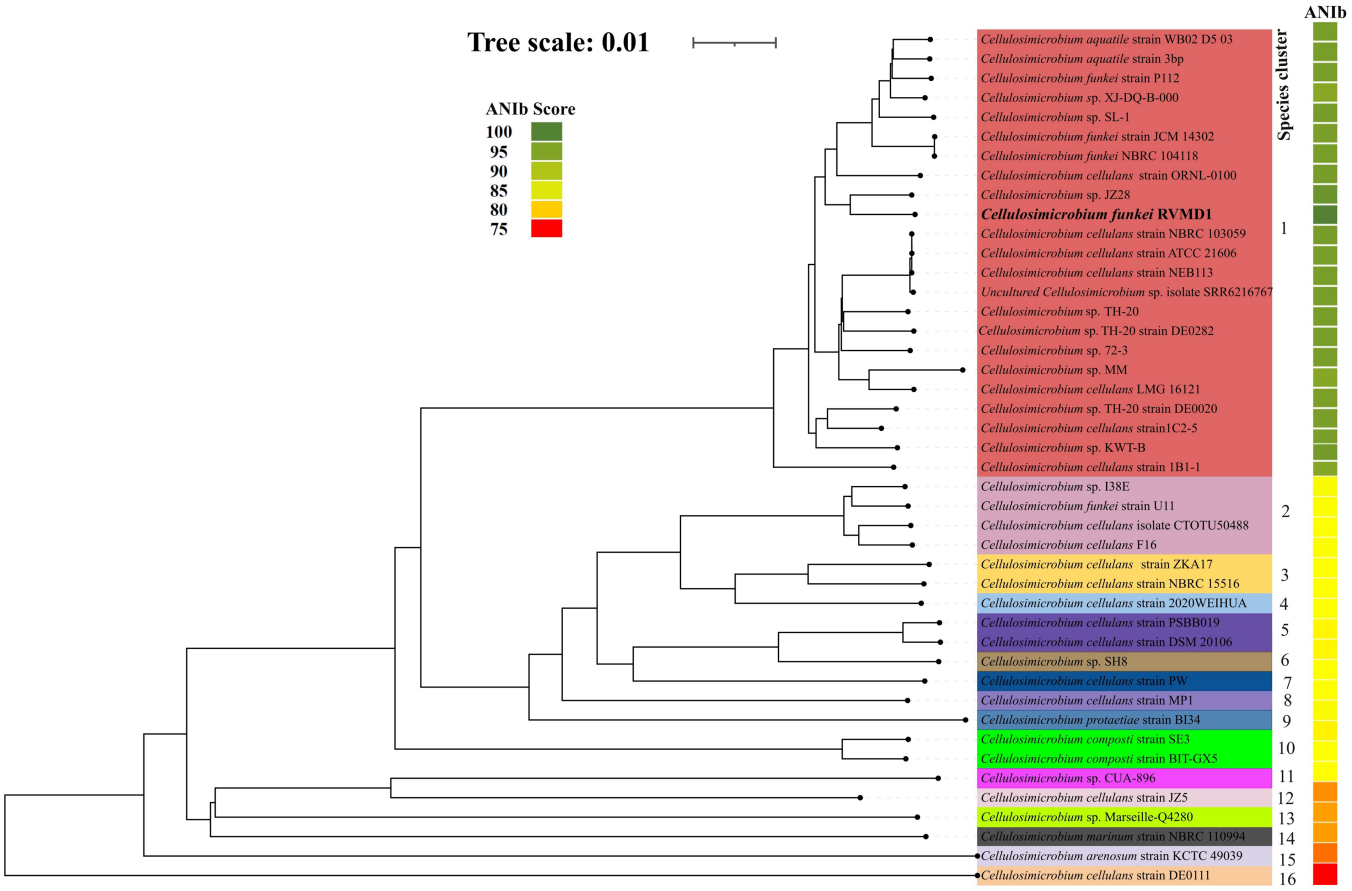
The genome tree inferred with FastME 2.1.6.1 from GBDP distances calculated from genome sequences. The branch lengths are scaled in terms of GBDP distance formula d*5*. The numbers above branches are GBDP pseudo-bootstrap support values >60% from 100 replications, with an average branch support of 96.1%. Rooted at the midpoint, this analysis includes 43 genomes from the *Cellulosimicrobium* genus, along with our isolate, *C. funkei* RVMD1, as referenced in NCBI. The color strips indicate the ANIb values for each species compared to our isolate, *C. funkei* RVMD1.

As indicated in Supplementary Table S4 and Figures 6, 7, *C. funkei* RVMD1 has a typical genome size for the genus (4,264,015 base pairs). Surprisingly, its protein counts of 4,449 greatly exceeds the genus average. This suggests a high coding density, as confirmed by the 90.27% value found in Table 2, according to MiGA (Rodriguez-R et al., 2018). Within the phylogenomic analysis, the genomes are categorized into 16 species clusters. Cluster 1 is of particular interest as it is linked to the *C. cellulans* group and includes the *C. funkei* RVMD1 isolate. This cluster is the most extensive, encompassing a variety of strains: *C.* sp. XJ-DQ-B-000, *C. cellulans* strain NEB113, *C. cellulans* strain NBRC 103059, *C. cellulans* strain ATCC 21606, *C.* sp. 72–3, *C.* sp. TH-20, *C. funkei* strain P112, *C. aquatile* strain 3 bp, an uncultured *C.* sp. isolate SRR6216767 MAG genomic, *C.* sp. TH-20 strain DE0020, *C.* sp. SL-1, *C.* sp. KWT-B, *C.* sp. JZ28, *C.* sp. TH-20 strain DE0282, *C. funkei* strain JCM 14302, *C. aquatile* strain WB02 D5 03, *C. cellulans* ORNL-0100, and *C. funkei* NBRC 104118. This cluster encompasses the majority of the genomes, indicating a close genetic relationship among the strains within cluster 1, and is likely indicative of their belonging to the same species group, a conclusion that is supported by ANIb values for all members of the cluster when compared with *C. funkei* RVMD1, which range from 97.5 to 98.49% (Chun et al., 2018). It is clear from the phylogenetic analysis that the most genetically close strain to *C. funkei* RVMD1 is *C.* sp. JZ28, a root endophytic bacterium from the desert plant *Panicum turgidum*, with genes for stress resistance, volatile production, biocontrol, and biopolymer breakdown (Eida et al., 2020).

Clusters 2 through 16 display a more streamlined composition compared to cluster 1, with clusters 4, 6, 7, 8, 9, 11, 12, 13, 14, 15, and 16 each uniquely containing a single strain. This distinct arrangement underscores the unique genetic differences that each of these strains has from others within the genus. In contrast, clusters 2, 3, 5, and 10 are characterized by multiple strains. Cluster 2 is a diverse group, including *C. cellulans* isolate CTOTU50488, *C. cellulans* F16, *C. funkei* strain U11, and *C.* sp. I38E. Cluster 3 is home to two strains of *C. cellulans*, namely NBRC 15516 and ZKA17, cluster 5 combines *C. cellulans* strain DSM 20106 with strain PSBB019. Cluster 10 is comprised of two strains of *C. composti* strains SE3 and BIT-GX5.

Moreover, a remarkable tight genomic consistency in G + C content within species of the same cluster is observed, with variations not exceeding 1%. This observation is in line with the findings of Meier-Kolthoff et al. (2014), which reported that within-species differences in G + C content are typically less than 1%. Such consistency in G + C content further supports the clustering method utilized by TYGS as an effective tool for identifying genetically coherent groups within the *Cellulosimicrobium* genus.

**Figure 8:**
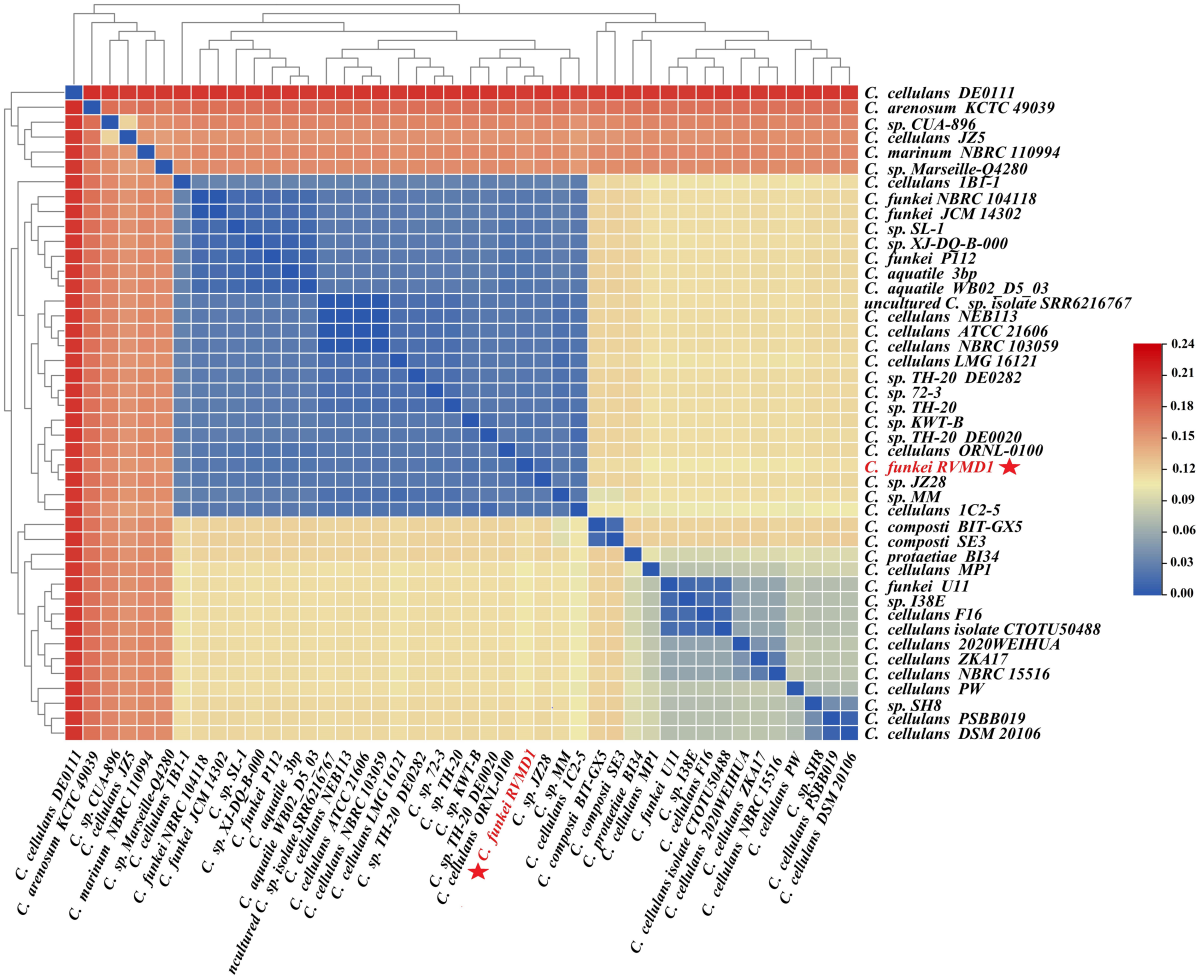
ANI/AAI matrix heatmap of *Cellulosimicrobium* strains with hierarchical clustering. This heatmap represents the all-*vs*-all comparison of Average Nucleotide Identity (ANI) and Average Amino Acid Identity (AAI) among various strains of *Cellulosimicrobium*, including *C. funkei* RVMD1. The color gradient from blue to red indicates the degree of genomic similarity, with blue representing higher similarity (closer species) and red indicating lower similarity. The dendrogram on both the x and y axes classify the strains based on genomic relatedness.

**Figure 9:**
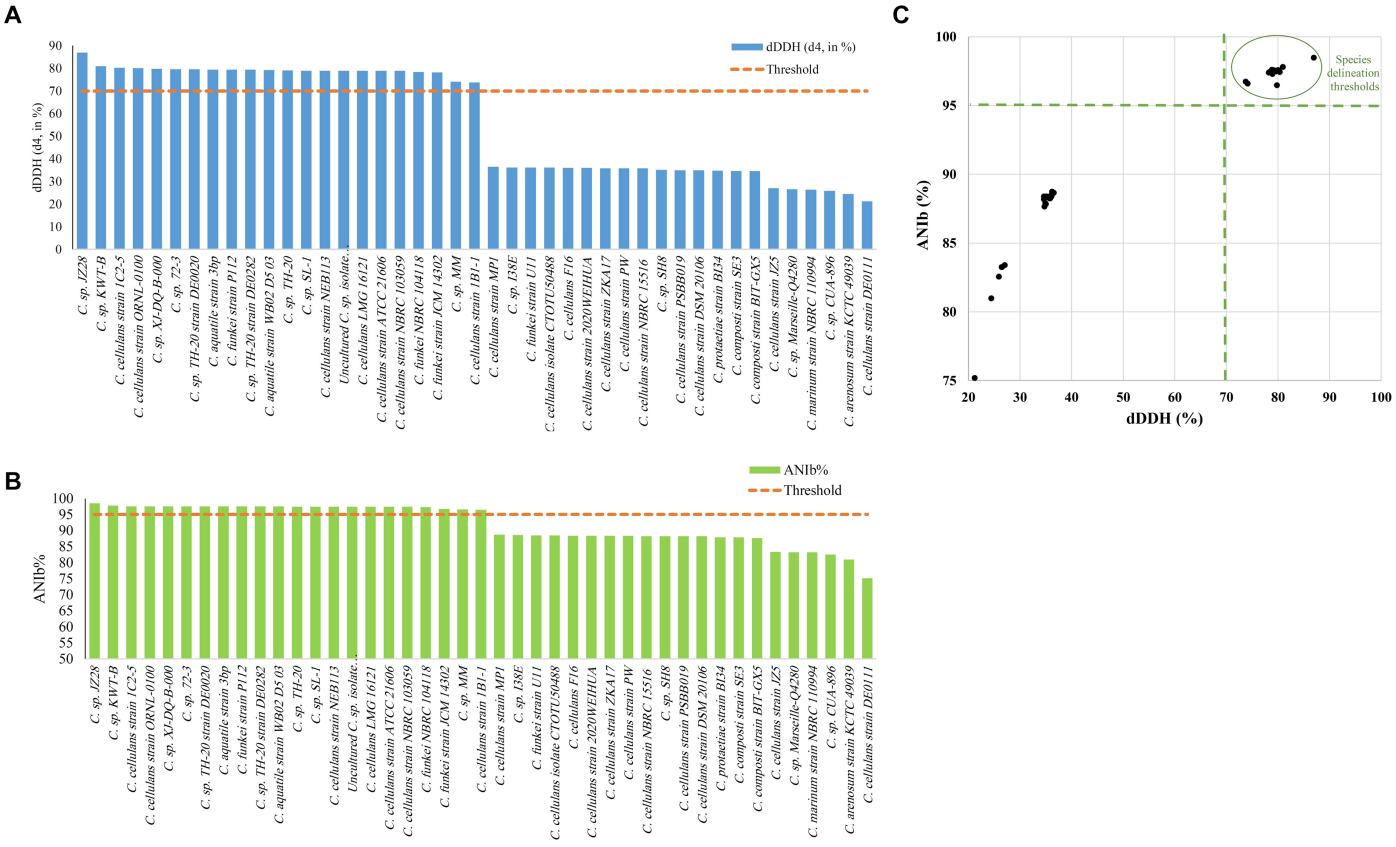
Delineating *C. funkei* RVMD1 among 43 genomes from the *Cellulosimicrobium* genus. **(A)** The digital DNA–DNA hybridization (dDDH) values were calculated using Genome-to-Genome Distance Calculator (GGDC) analysis, utilizing a 70% species delineation threshold. **(B)** The Average Nucleotide Identity by BLAST (ANIb) analysis, with a (>95%) species delineation threshold. **(C)** A scatter plot of pairwise digital DNA–DNA hybridization (dDDH) vs. ANIb values for *C. funkei* RVMD1 against the 43 genomes highlights matches that exceed the species thresholds, clarifying the taxonomic standing of *C. funkei* RVMD1.

The ANI/AAI matrix heatmap (Figure 8), providing all-*vs*-all genomic distance estimates, strongly supports the clustering and topologies seen in the *Cellulosimicrobium* phylogenomic tree. Both analyses use ANI and AAI metrics for comprehensive similarity assessment. This agreement is visually clear the 43 species, including our isolate, form 16 cluster. Strains closely grouped on the tree share higher ANI/AAI values (blue on the heatmap), while those more distant shift toward red, further confirming their phylogenetic separation. ANIb and dDDH analyses (Figure 9) reveal significant genomic variation within the *Cellulosimicrobium* genus. Strains within cluster 1 (including the *C. cellulans* group) exhibit high ANIb (>95%) and dDDH (>70%) values (Chun et al., 2018), indicating strong similarity and justifying their shared species cluster. Other clusters show lower values, highlighting their distinction from cluster 1 and the differences between *C. funkei* RVMD1 and the *C. cellulans* group. This variation underscores the need to carefully analyze species boundaries and potentially revise classification criteria within the genus.

The aforementioned analyses make it clear that studied *C. cellulans* strains (18/43) are distributed across various species clusters, suggesting potential inaccuracies in current taxonomy assignments and raising the possibility that some strains may represent divergent species. In response to this observation, we conducted all-*vs*-all OrthoANI and ANIb analyses among all *C. cellulans* species.

**Figure 10:**
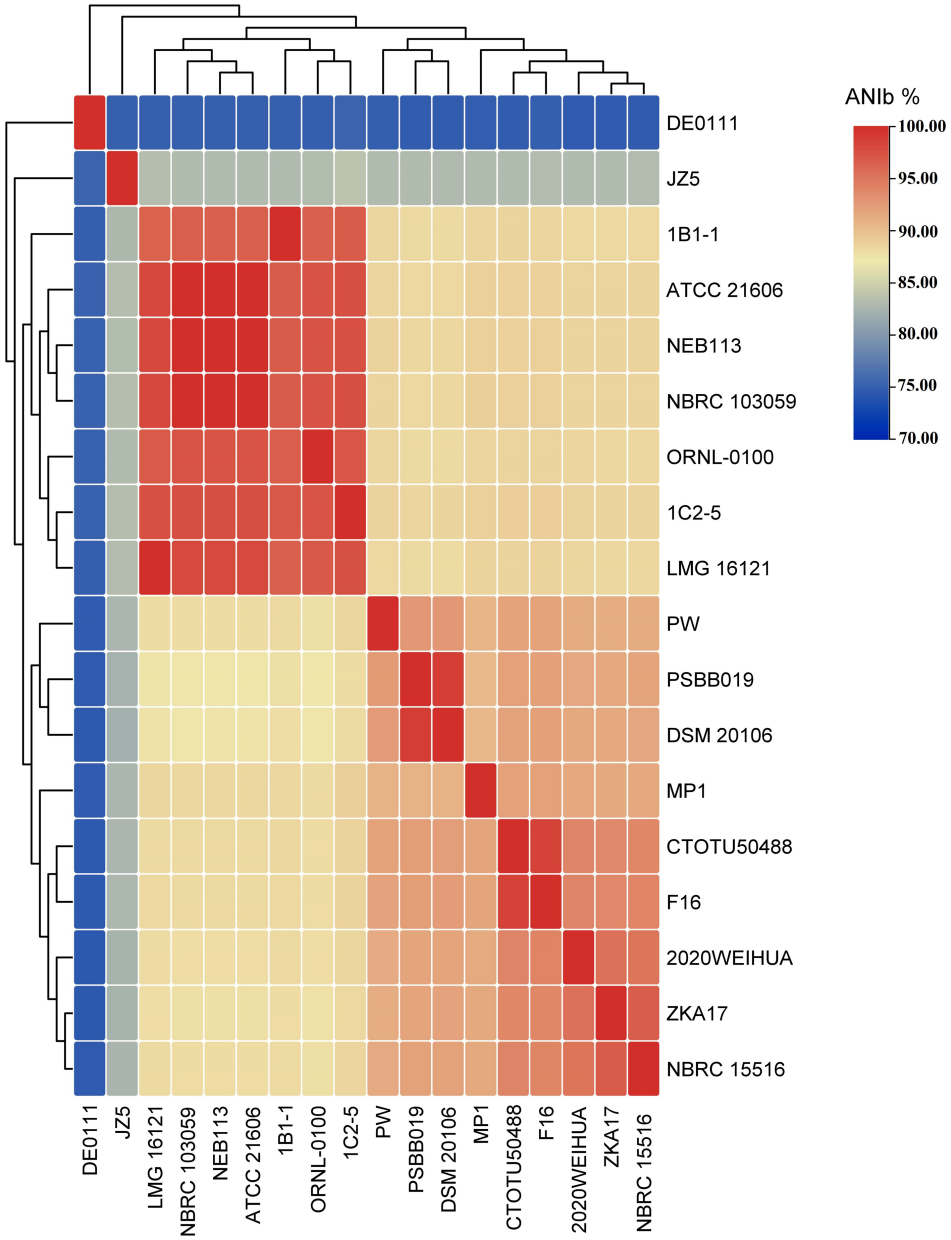
All-*vs*-all ANIb heatmap of all *C. cellulans* species from different species clusters.

Figure 10, Supplementary Figure S6 and Supplementary Table S5, demonstrate a clear division of *C. cellulans* into nine different genomospecies, indicating a shift from a singular species classification to potentially up to nine distinct species. This is particularly evident when examining the pairwise ANIb indices against the reference strain ORNL-0100. Strains including NEB113, NBRC 103059, ATCC 21606, LMG 16121, 1C2-5, and 1B1-1 show ANIb values that exceed the 95–96% threshold for species designation (Chun et al., 2018; Wu et al., 2020), ranging from 96.72 to 97.42%. Their close proximity to the reference strain confirms that only these strains likely should be designated as “*cellulans*.” Our findings support the results of a similar comparative study, although that study examined fewer species (Aviles and Kyndt, 2021). The remaining strains, including NBRC 15516 and ZKA17, DSM 20106 and PSBB019, isolate CTOTU50488 and F16, 2020WEIHUA, MP1, PW, DE0111, and JZ5, form eight distinct genomospecies. These clusters exhibit ANIb values, much lower than the threshold, ranging from 75.24 to 88.71% when compared to both the reference strain and each other (Meier-Kolthoff et al., 2013). This strongly supports the hypothesis of species misidentification and suggests the need for taxonomic revision within the *Cellulosimicrobium* genus. This revision could potentially double the number of official *Cellulosimicrobium* species.

### 3.5 Comparative phylogenomic analysis

**Figure 11:**
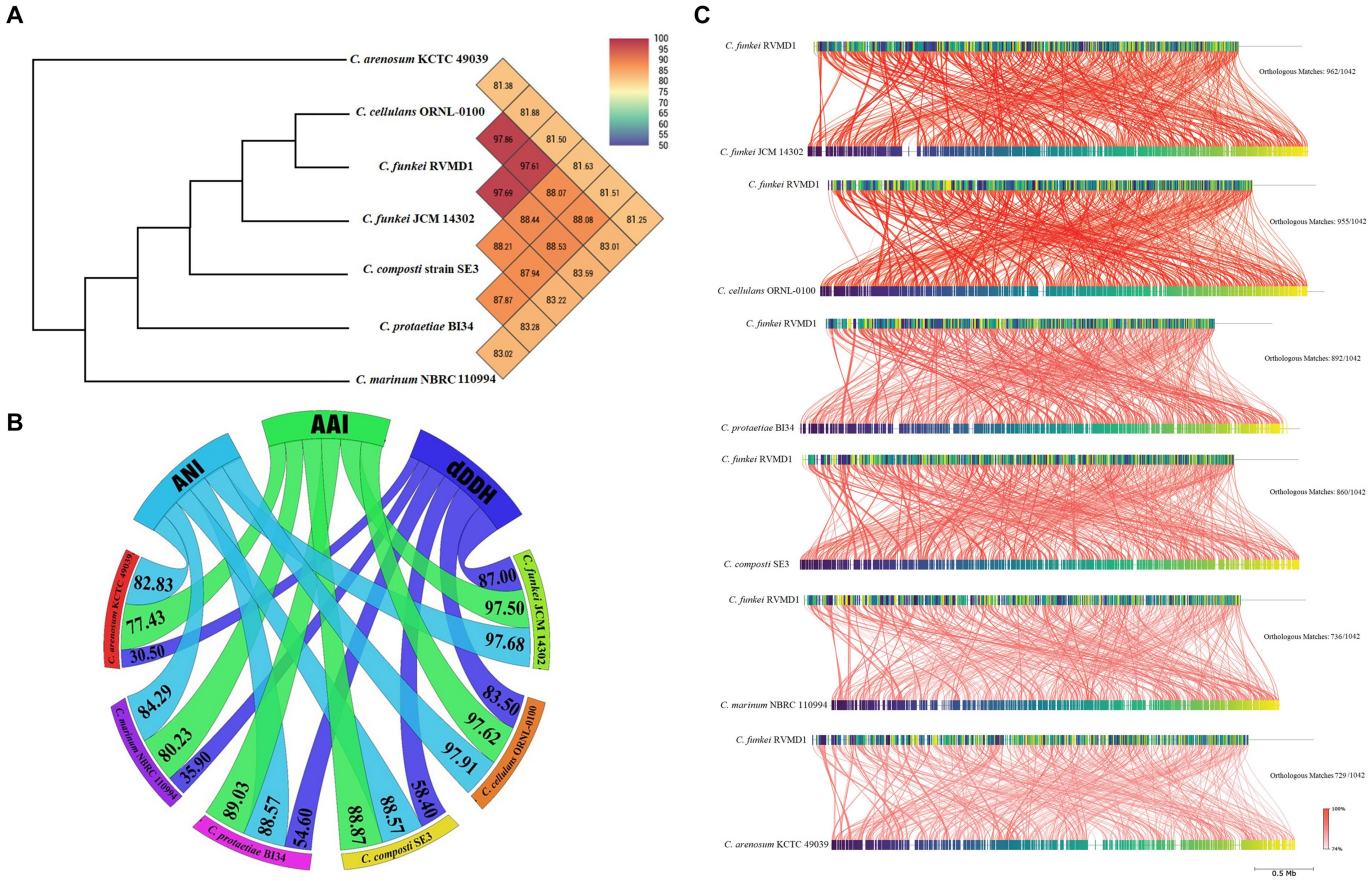
OrthoANI, OGRI, and whole-genome similarity relationships between *C. funkei* RVMD1 and reference *Cellulosimicrobium* species. **(A)** Heatmap of Orthologous Average Nucleotide Identity (OrthoANI) with an ANI phylogenetic tree constructed based on OrthoANI values calculated using OAT software. **(B)** Chord diagram illustrating Orthogonal Genomic Relationship Index (OGRI) values, including AAI, ANI, and dDDH. **(C)** Whole-genome similarity analysis using FastANI, showing conserved genomic regions, orthologous matches, and evolutionary relationships.

As mentioned previously, the *Cellulosimicrobium* genus contains six valid, distinct species. Each species has a designated reference strain: *C. protaetiae* BI34*, C. cellulans* ORNL-0100, *C. funkei* JCM 14302, *C. marinum* NBRC 110994, *C. composti* SE3 and *C. arenosum* KCTC 49039. For in-depth genome analysis, these reference strains were chosen to facilitate a comparative study with our isolate, *C. funkei* RVMD1. As anticipated, OGRI metrics (OrthoANI, ANI, AAI, and dDDH), illustrated in Figures 11A,B, confirm the systematic position of RVMD1 as a species within the *C. cellulans* group. Of note, *C. cellulans* ORNL-0100 and *C. funkei* JCM 14302 demonstrated the highest OrthoANI, ANI, AAI, and dDDH values with *C. funkei* RVMD1, exceeding the species cutoff delineation (Chun et al., 2018). OrthoANI offers a reliable and faster way to determine average nucleotide identity (ANI) for classifying organisms. It strongly aligns with results from traditional ANI methods (using BLASTn) (Lee et al., 2016). In contrast, other reference species demonstrated significantly lower OrthoANI (81.88–88.53%), ANI (82.83–88.57%), AAI (77.43–89.03%), and dDDH (30.50–58.40%) values. To further assess genome similarity, a detailed whole-genome similarity analysis was conducted using the FastANI 1.3.3 tool, as shown in Figure 11C. This analysis provides a comprehensive visualization of genomic similarities between *C. funkei* RVMD1 and the reference strains. Each red line in the figure represents reciprocal mappings, indicating conserved sequences and evolutionary relationships, with varying line intensity corresponding to different ANI values. Orthologous matches (e.g., 962/1,042 with *C. funkei* JCM 14302 and 729/1,042 with *C. arenosum* KCTC 49039) reflect the degree of genomic similarity. Consistent with the OGRI results, *C. cellulans* ORNL-0100 and *C. funkei* JCM 14302 exhibited the highest degree of similarity, supporting their close relationship with our isolate, *C. funkei* RVMD1, while the other reference species showed more divergence. Here, the calculated OGRI values and whole-genome similarity analysis based on the WGS demonstrate that species boundaries are distinctly observed between all reference species and our isolate *C. funkei* RVMD1, except for *C. cellulans* ORNL-0100 and *C. funkei* JCM 14302, potentially necessitating their reclassification (Supplementary Figure S7).

**Figure 12:**
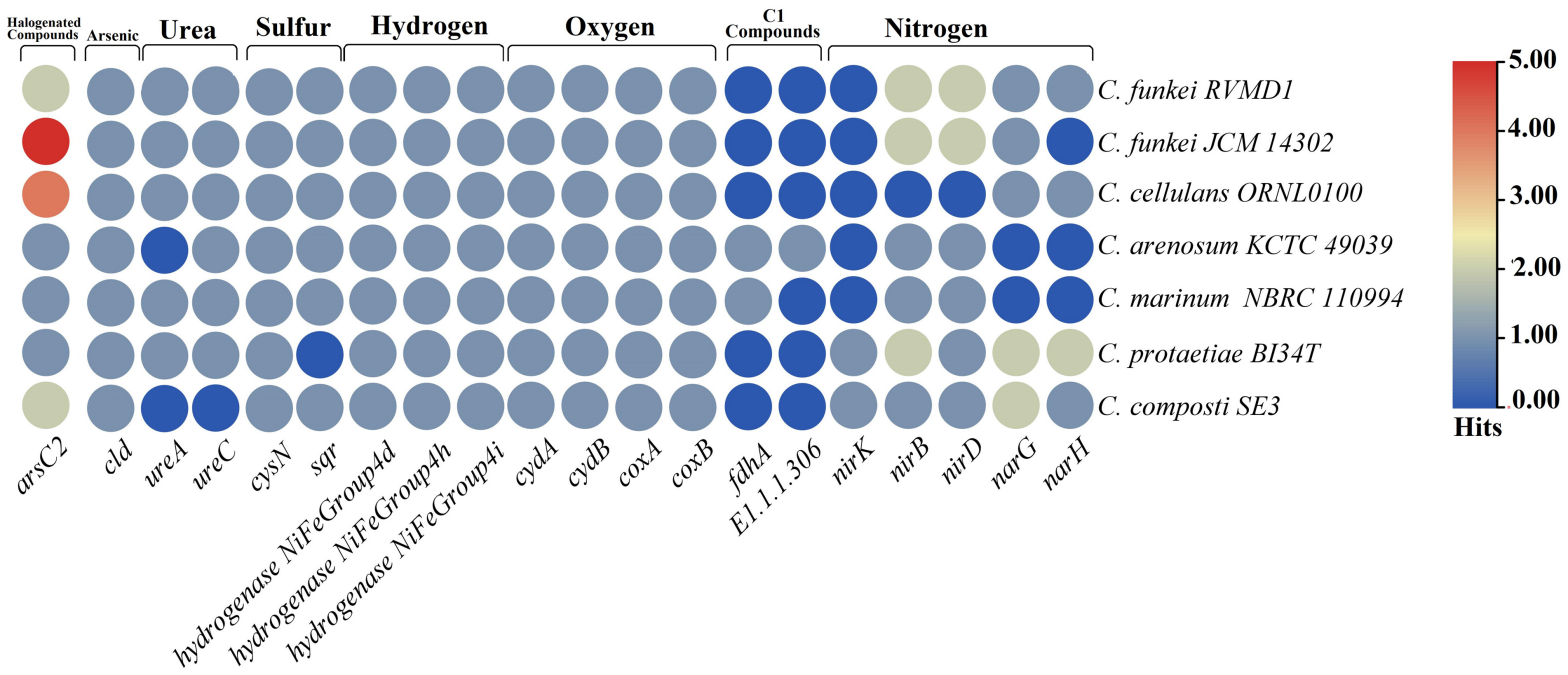
Abundance of MicroTrait bioelement family genes in *C. funkei* RVMD1 and the reference species of the genus *Cellulosimicrobium*. The heatmap illustrates the distribution and relative abundance of gene families involved in key bioelement cycles, including halogenated compounds, arsenic, urea, sulfur, hydrogen, oxygen, C1 compounds, and nitrogen.

The analysis of bioelement cycling genes within the *C. funkei* RVMD1 genome (Figure 12), as conducted through microTrait (Karaoz and Brodie, 2022), which facilitates the examination of ecologically relevant traits directly from microbial genome sequences, has unveiled a microbe that is likely capable of exhibiting remarkable metabolic adaptability. This adaptability is particularly suited to the harsh conditions found in desert rock varnish. The genomic analysis reveals a potential for denitrification (*narG*, *narH*, *nirB*, *nirD*), oxygen utilization (*cydA*/*cydB*, *coxA*/*coxB*), sulfur cycling (*cysN*, *sqr*), and diverse hydrogen metabolism (*NiFe* Group 4d/h/i hydrogenases). Such versatility underscores the microbe’s ability to navigate varying oxygen conditions and resource availability efficiently. Furthermore, the presence of urease genes (*ureA*, *ureC*) underscores the microbe’s capability to utilize urea as a nitrogen source, enhancing its adaptability. Despite a substantial number of contracted genes in response to arsenic, the presence of arsenic reduction potential (*arsC2*) and chlorite dismutase (*cld*) genes indicates that this organism adapts to various chemical stressors. This adaptation is crucial in its desert rock varnish habitat, where arsenic may occur as a component (Sims et al., 2022). The full nitrogen and sulfur metabolism gene comparisons are shown as a heatmap in Supplementary Figure S8, highlighting differences between *C. funkei* RVMD1 and other *Cellulosimicrobium* strains. For the CAZyme analysis, CAZyme families (GH, GT, PL, CE, AA, and CBM) were compared using Galaxy Protologger (Hitch et al., 2021). As shown in Supplementary Table S6, *C. funkei* RVMD1 has 288 CAZymes, slightly more than *C. cellulans* ORNL-0100 and *C. funkei* JCM 14302. This diversity suggests a broader carbohydrate degradation capability within this group, supporting adaptation to challenging environments.

**Figure 13:**
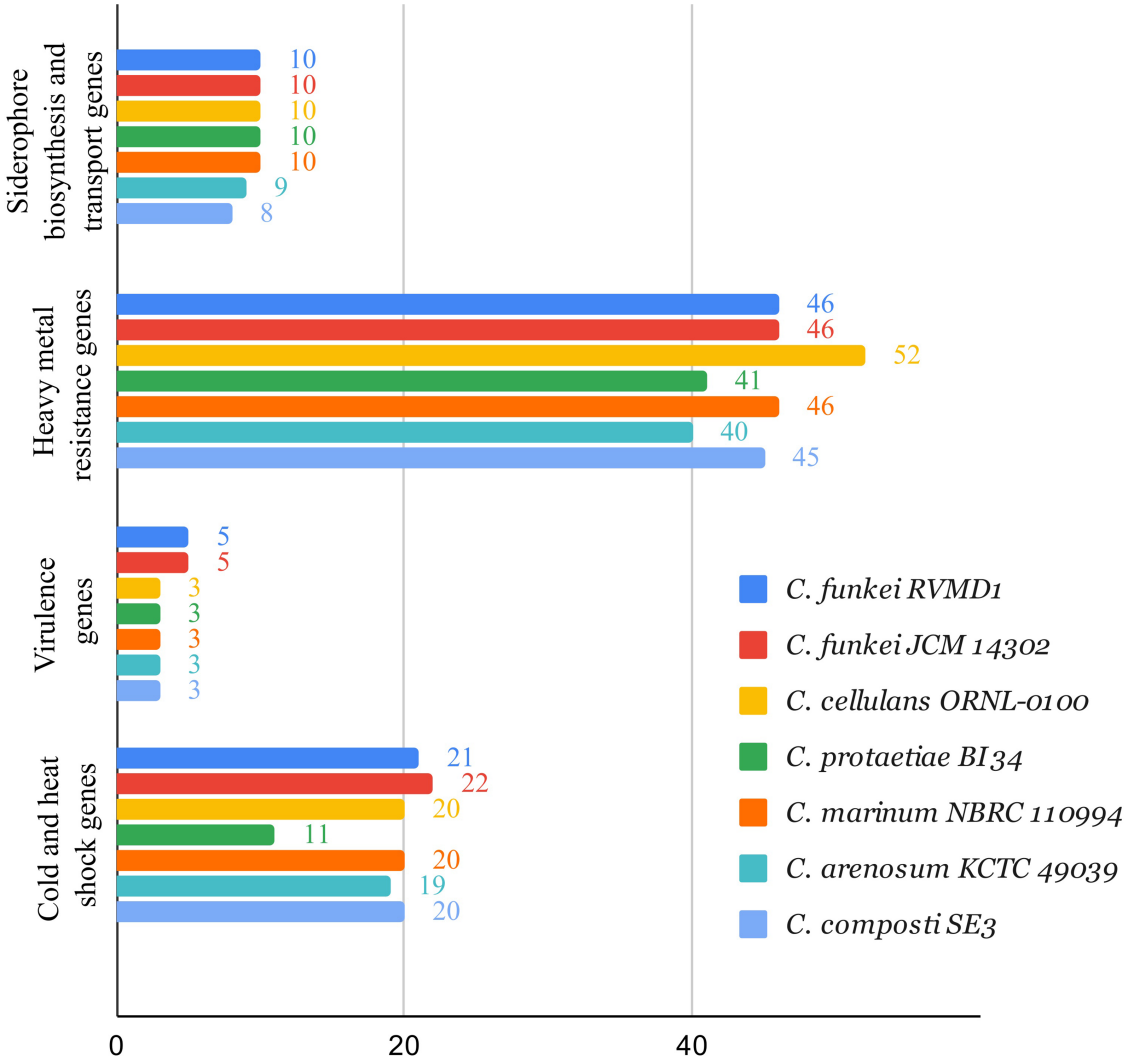
Comparison of siderophore biosynthesis, heavy metal resistance, virulence, and cold and heat shock genes in *C. funkei* RVMD1 against other reference strains across the *Cellulosimicrobium* genus, using Bacterial Bioinformatics Resource Center (BV-BRC) annotation.

Based on the BV-BRC annotation (Olson et al., 2023), as shown in Figure 13, the analyzed set of genes for siderophore biosynthesis, heavy metal resistance, virulence, and cold and heat shock genes in *C. funkei* RVMD1, compared to other reference strains across the *Cellulosimicrobium* genus, are largely similar with some variations. *C. funkei* RVMD1 has 46 heavy metal resistance genes, close to *C. funkei* JCM 14302 and *C. marinum* NBRC 110994, but fewer than *C. cellulans* ORNL-0100 (52). It contains 10 siderophore biosynthesis genes, aligning with most strains, and has five virulence genes, while others have three. For cold and heat shock genes, RVMD1 has 21, slightly fewer than JCM 14302 (22). These findings suggest that the analyzed set of genes across the tested genomes is relatively similar, with *C. funkei* RVMD1 showing only a few differences. This indicates that RVMD1 has a balanced set of genes that are almost conserved among this genus, potentially supporting its adaptability in diverse environments. This adaptability aligns with the variety of habitats from which *Cellulosimicrobium* species have been isolated, including soil, clinical samples, and marine sponges (Brown et al., 2006; Yoon et al., 2007; Li, 2009; Antony et al., 2009).

Moreover, bacterial isolates from rock varnish in the same study area have exhibited exceptional tolerance to iron (II) and various heavy metals, including lead, copper, chromium, silver, and mercury (Alnaimat et al., 2017). In a recent study, a similar strain, *C. funkei* AR6, has been effectively used in the bioremediation of heavy metal-contaminated environments (Karthik et al., 2021). This finding suggests potential benefits of our isolate, *C. funkei* RVMD1, in such applications and underscores the broader environmental adaptability of the *Cellulosimicrobium* genus.

**Figure 14:**
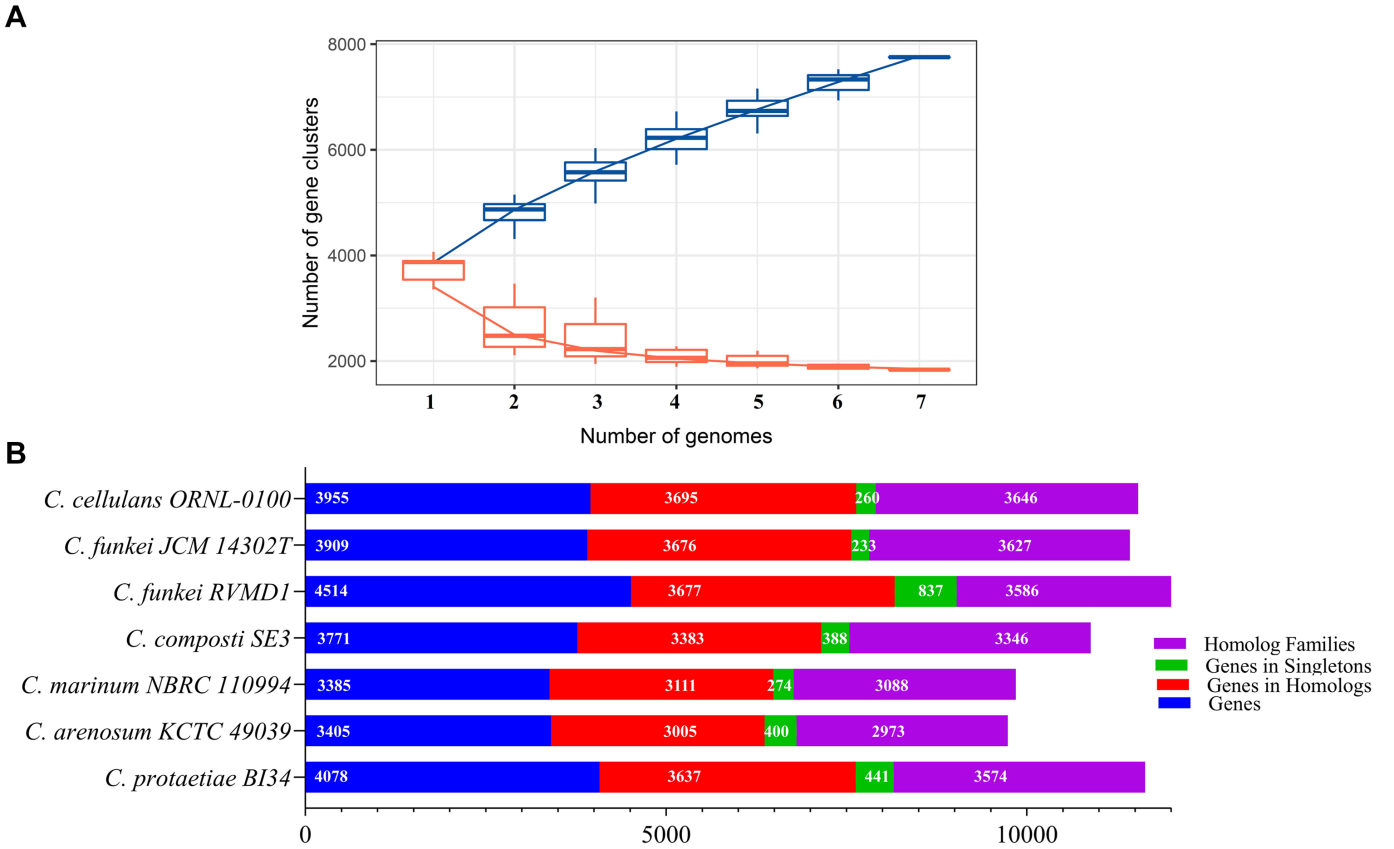
Pangenome analysis of RVMD1 and six *Cellulosimicrobium* reference species. **(A)** Rarefaction curves comparing pan-gene (blue) and core gene (orange) clusters, generated using IPGA. **(B)** Comparative pangenome analysis constructed using Build Pangenome with OrthoMCL - v2.0 on the KBase platform.

**Figure 15:**
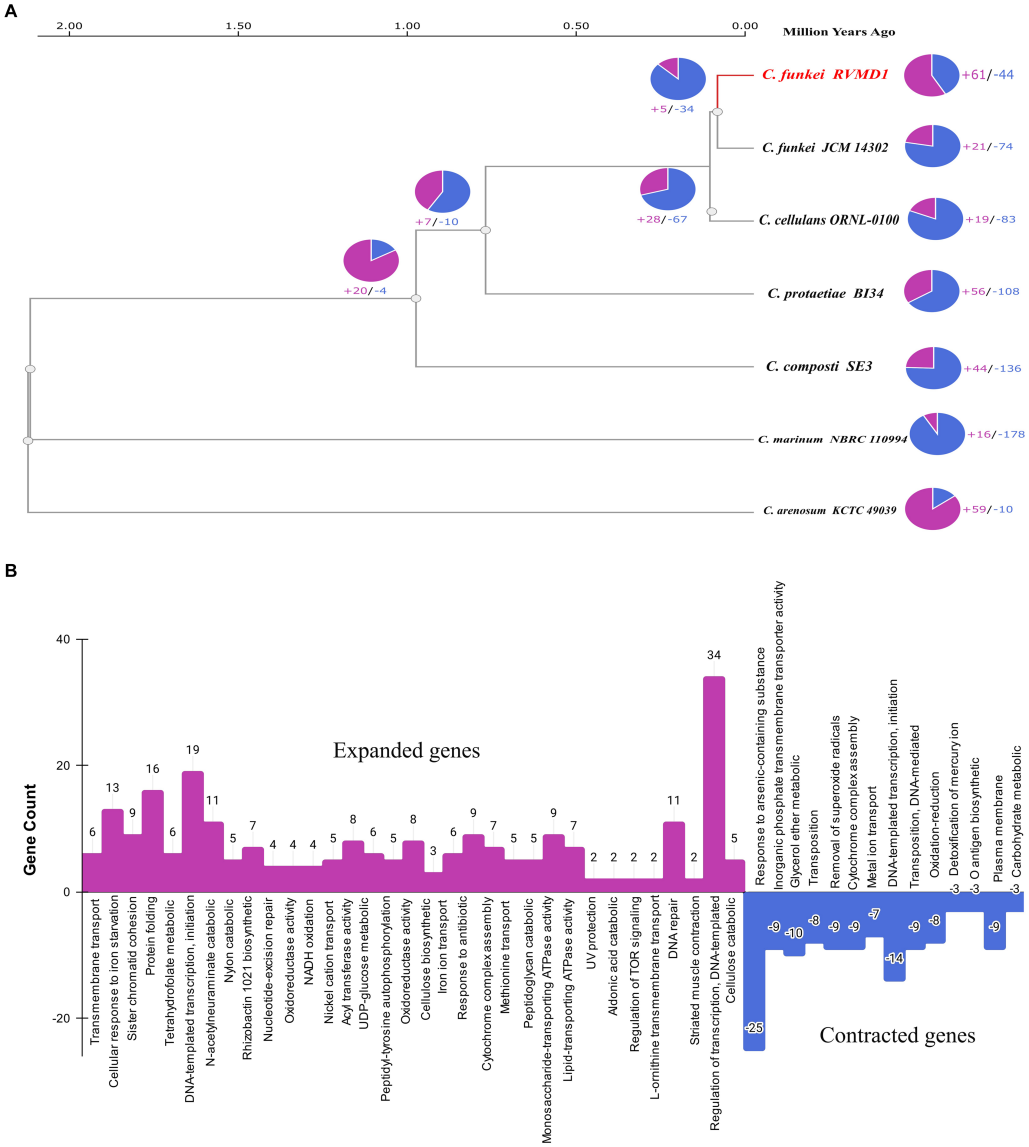
**(A)** Phylogenetic and gene family dynamics in *C. funkei* RVMD1 and reference genomes of the *Cellulosimicrobium* genus. The ultrametric phylogenetic tree based on single-copy genes illustrates the evolutionary relationships and distances among the species in Mya, complemented by pie charts that quantify gene family expansions (purple) and contractions (blue). **(B)** The bar chart visualizes Gene Ontology (GO) annotations for expanded (in purple) and contracted (in blue) gene families in *C. funkei* RVMD1.

A pangenomic analysis of *Cellulosimicrobium* six reference species in addition RVMD1 isolate was performed, using Build Pangenome with OrthoMCL - v2.0 on the KBase platform and IPGA for rarefaction curve analysis (Arkin et al., 2018; Liu et al., 2022). The rarefaction curves (Figure 14A) show that the pan-genome has 7,754 gene clusters, indicating high genetic diversity, while the core genome has 1,835 gene clusters, representing conserved genes. The pan-genome continues to grow with more genomes, indicating it is open, while the core genome’s early plateau shows a stable set of shared genes. These findings set the stage for our refined analysis (Figure 14B), which revealed a genome comprising 4,514 genes for the *C. funkei* RVMD1 isolate, with 3,677 genes positioned within homologous families and 837 identified as unique singletons. This places RVMD1 at the forefront in terms of total gene count, genes within homologous families, and singleton genes, highlighting its significant genetic distinctiveness within the *Cellulosimicrobium* pangenome. Additionally, RVMD1 exhibits 3,586 homolog families, the highest number among the species analyzed, further emphasizing its unique genetic contribution to the genus. Comparative analysis revealed varied genetic compositions among the species, highlighting the diversity within the genus. The discovery of 1,835 core gene families shared across all genomes underscores a fundamental genomic backbone critical for the biological and evolutionary coherence of the *Cellulosimicrobium* genus. The 837 singleton genes of RVMD1 signal its specialized ecological niches and evolutionary adaptations, which could be further explained by the gene family dynamics in this isolate, as illustrated in Figure 15.

The phylogenetic gene family dynamics represented in Figure 15A and the gene ontology annotations in Figure 15B collectively explore the evolutionary strategies that could shape the genomic profile of *C. funkei* RVMD1, especially when considered in the context of its extreme habitat in the rock varnish of the Ma’an desert. This environment, characterized by intense solar irradiation with peak values of 6,670–6,780 Wh/m^2^/day (Alnaimat et al., 2017; Alrwashdeh et al., 2018), subjects the microbial community to extreme UV light pressure and temperature fluctuations, alongside nutrient scarcity. An ultrametric phylogenetic tree (Figure 15A) reveals the evolutionary history of *C. funkei* RVMD1 along with reference genomes of the *Cellulosimicrobium* genus. The associated pie charts quantify the gene family expansions and contractions, providing a visual representation of genomic flux. *C. funkei* RVMD1 shows a significant number of gene family expansions, which may be indicative of adaptive evolution in response to the challenges of its habitat. In Figure 15B, the bar chart details the specific functional categories of these expanded and contracted gene families, based on Gene Ontology (GO) annotations. The considerable expansion of genes involved in transcription regulation, cellular response to iron ion starvation, and protein folding again points to a genetic reinforcement of pathways that might confer resilience to environmental stresses. The striking expansion in genes associated with DNA repair, such as those involved in nucleotide-excision repair, pre-cleavage complex stabilization, and regulation of transcription, DNA-templated, is consistent with an adaptive response to the high levels of DNA damage that would be expected from intense UV radiation (Ghosh et al., 2023). On the other hand, genes related to the response to arsenic-containing substances have undergone significant contraction, which could reflect a reduced selective pressure in this aspect of the environment, or possibly a trade-off where resources are reallocated to more critical survival functions. Overall, the genome of *C. funkei* RVMD1 demonstrates how gene family expansion and contraction, a critical mechanism in adaptation and speciation (Sharpton et al., 2009), could drive adaptation to harsh environments.

While this study provides valuable insights into the genome of *C. funkei* RVMD1, several limitations are important to acknowledge. First, even though the high number of contigs (857) in the genome sequence may affect the assembly quality, the overall results are still considered sufficiently accurate given the high coverage and quality metrics obtained. Second, a purely genomic approach may not fully represent the organism’s complex ecological interactions. Third, despite rigorous antiseptic measures during sampling, the possibility remains that the isolate may have originated from external sources such as adjacent soil or air, rather than being definitively endogenous to the rock varnish layer. Finally, while genomic analysis suggests potential antibiotic resistance and virulence factors, phenotypic studies are needed to confirm their real-world implications for human health and disease.

## 4 Conclusion

This study offers vital insights into microbial diversity within desert rock varnish. Through the isolation and genomic characterization of *C. funkei* isolate RVMD1, we found that the current systematics within the *Cellulosimicrobium* genus need to be reassessed, as 16S rRNA gene sequencing is not sufficient for species delineation. Our comprehensive genomic analysis confirms the taxonomic position of RVMD1 and suggests that it has evolved specific adaptations for survival in harsh desert environments. The presence of genes associated with antimicrobial resistance and potential virulence factors in the RVMD1 genome raises concerns for human health. This study highlights the importance of integrating genomic analyses into taxonomic studies and calls for a reassessment of the taxonomic framework within the *Cellulosimicrobium* genus. Future research should explore the ecological roles and biotechnological potentials of this microbial community.

## Data Availability

The genomic data for *C. funkei* RVMD1 were submitted to GenBank under the BioProject accession number PRJNA987486. The specific BioSample associated with this project is SAMN35982307. This Whole Genome Shotgun project has been deposited at DDBJ/ENA/GenBank under the accession JAUECY000000000. The version described in this paper is version JAUECY010000000. The raw fastq files from genome sequencing were deposited in the NCBI Sequence Read Archive (SRA) under accession number SRR25069828. Additionally, the 16S rRNA gene sequence is accessible under the GenBank accession number OR570906.

## Footnotes

^1^ https://www.ncbi.nlm.nih.gov/datasets/genome/?taxon=157920

^2^ www.EzBioCloud.net

^3^ www.protologger.de

^4^ https://itol.embl.de

^5^KBase, http://kbase.us.

^6^ www.bv-brc.org

^7^ www.microbial-genomes.org

^8^ www.ezbiome.app

^9^ https://proksee.ca/

^10^ https://rast.nmpdr.org/seedviewer.cgi

^11^ https://www.ncbi.nlm.nih.gov/datasets/genome/?taxon=157920&typical_only=true

^12^ https://tygs.dsmz.de/

^13^ www.jspecies.ribohost.com/jspeciesws/#analyse

^14^ http://ggdc.dsmz.de/ggdc.php

^15^ http://enve-omics.ce.gatech.edu/g-matrix/

^16^ http://ekhidna2.biocenter.helsinki.fi/AAI/

^17^ https://www.ezbiocloud.net/tools/orthoani

^18^ https://circos.ca/

^19^ https://nmdc.cn/ipga/

^20^ https://orthovenn3.bioinfotoolkits.net

## References

[ref1] Alnaimat S. Shattal S. A. Althunibat O. Alsbou E. Amasha R. H. (2017). Iron (II) and other heavy-metal tolerance in bacteria isolated from rock varnish in the arid region of Al-jafer basin, Jordan. Biodiversitas 18, 1250–1257. doi: 10.13057/biodiv/d180350

[ref2] Alrwashdeh S. S. Alsaraireh F. M. Saraireh M. A. (2018). Solar radiation map of Jordan governorates. Int. J. Eng. Sci. Technol. 7, 1664–1667. doi: 10.14419/ijet.v7i3.15557

[ref3] Antony R. Krishnan K. P. Thomas S. Abraham W. P. Thamban M. (2009). Phenotypic and molecular identification of *Cellulosimicrobium cellulans* isolated from Antarctic snow. Antonie Van Leeuwenhoek 96, 627–634. doi: 10.1007/s10482-009-9377-9, PMID: 19760124

[ref4] Arkin A. P. Cottingham R. W. Henry C. S. Harris N. L. Stevens R. L. Maslov S.. (2018). KBase: the United States Department of Energy systems biology knowledgebase. Nat. Biotechnol. 36, 566–569. doi: 10.1038/nbt.4163 29979655 PMC6870991

[ref5] Ashburner M. Ball C. A. Blake J. A. Botstein D. Butler H. Cherry J. M.. (2000). Gene ontology: tool for the unification of biology. The Gene Ontology Consortium. Nat. Genet. 25, 25–29. doi: 10.1038/75556, PMID: 10802651 PMC3037419

[ref6] Aviles F. A. Kyndt J. A. (2021). Cellulosimicrobium fucosivorans sp. nov., isolated from San Elijo Lagoon, contains a fucose metabolic pathway linked to carotenoid production. Arch. Microbiol. 203, 4525–4538. doi: 10.1007/s00203-021-02443-y, PMID: 34148152

[ref7] Aziz R. K. Bartels D. Best A. A. DeJongh M. Disz T. Edwards R. A.. (2008). The RAST server: rapid annotations using subsystems technology. BMC Genomics 9:75. doi: 10.1186/1471-2164-9-75, PMID: 18261238 PMC2265698

[ref8] Brown J. M. Steigerwalt A. G. Morey R. E. Daneshvar M. I. Romero L. J. McNeil M. M. (2006). Characterization of clinical isolates previously identified as *Oerskovia turbata*: proposal of *Cellulosimicrobium funkei* sp. nov. and emended description of the genus Cellulosimicrobium. Int. J. Syst. Evol. Microbiol. 56, 801–804. doi: 10.1099/ijs.0.63882-0, PMID: 16585698

[ref9] Chaumeil P. A. Mussig A. J. Hugenholtz P. Parks D. H. (2019). GTDB-Tk: a toolkit to classify genomes with the genome taxonomy database. Bioinformatics 36, 1925–1927. doi: 10.1093/bioinformatics/btz848, PMID: 31730192 PMC7703759

[ref10] Chen C. Wu Y. Li J. Wang X. Zeng Z. Xu J.. (2023). TBtools-II: a “one for all, all for one” bioinformatics platform for biological big-data mining. J. Mol. Plant. 16, 1733–1742. doi: 10.1016/j.molp.2023.09.010 37740491

[ref11] Chun J. Oren A. Ventosa A. Christensen H. Arahal D. R. da Costa M. S.. (2018). Proposed minimal standards for the use of genome data for the taxonomy of prokaryotes. Int. J. Syst. Evol. Microbiol. 68, 461–466. doi: 10.1099/ijsem.0.002516, PMID: 29292687

[ref1002] Culotta V. C. Wildeman A. S. (2021). Shining light on photosynthetic microbes and manganese-enriched rock varnish. Proc. Natl. Acad. Sci. USA. 118. doi: 10.1073/pnas.2109436118, PMID: 34183441 PMC8285942

[ref12] Davis J. J. Gerdes S. Olsen G. J. Olson R. Pusch G. D. Shukla M.. (2016). PATtyFams: protein families for the microbial genomes in the PATRIC database. Front. Microbiol. 7:118. doi: 10.3389/fmicb.2016.00118, PMID: 26903996 PMC4744870

[ref13] Delport J. Wakabayashi A. T. Anantha R. V. Lannigan R. John M. McCormick J. K. (2014). Cellulosmicrobium cellulans isolated from a patient with acute renal failure. JMM Case Rep. 1:000976. doi: 10.1099/jmmcr.0.000976

[ref14] Eida A. A. Bougouffa S. Alam I. Saad M. M. Hirt H. (2020). Complete genome sequence of the endophytic bacterium Cellulosimicrobium sp. JZ28 isolated from the root endosphere of the perennial desert tussock grass Panicum turgidum. Arch. Microbiol. 202, 1563–1569. doi: 10.1007/s00203-020-01859-2, PMID: 32172289

[ref15] Elarabi N. I. Halema A. A. Abdelhadi A. A. Henawy A. R. Samir O. Abdelhaleem H. A. R. (2023). Draft genome of *Raoultella planticola*, a high lead resistance bacterium from industrial wastewater. AMB Express 13:14. doi: 10.1186/s13568-023-01519-w, PMID: 36715862 PMC9885416

[ref16] Ewels P. Magnusson M. Lundin S. Kaller M. (2016). MultiQC: summarize analysis results for multiple tools and samples in a single report. Bioinformatics 32, 3047–3048. doi: 10.1093/bioinformatics/btw354, PMID: 27312411 PMC5039924

[ref17] Ghosh S. Banerjee S. Sengupta A. Peddireddy V. Mamillapalli A. Banerjee A.. (2023). “Survival and adaptation strategies of microorganisms in the extreme radiation,” in Bacterial survival in the hostile environment. eds. Kumar A. Tenguria S. (Cambridge, Massachusetts, USA: Academic Press), 219–229.

[ref1003] Grant J. R. Enns E. Marinier E. Mandal A. Herman E. K. Chen C.-y.. (2023). Proksee: in-depth characterization and visualization of bacterial genomes. Nucl. Acids. Res. 51, W484–W492. doi: 10.1093/nar/gkad326 37140037 PMC10320063

[ref18] Gurevich A. Saveliev V. Vyahhi N. Tesler G. (2013). QUAST: quality assessment tool for genome assemblies. Bioinformatics 29, 1072–1075. doi: 10.1093/bioinformatics/btt086, PMID: 23422339 PMC3624806

[ref19] Ha S. M. Kim C. K. Roh J. Byun J. H. Yang S. J. Choi S. B.. (2019). Application of the whole genome-based bacterial identification system, TrueBac ID, using clinical isolates that were not identified with three matrix-assisted laser desorption/ionization time-of-flight mass spectrometry (MALDI-TOF MS) systems. Ann. Lab. Med. 39, 530–536. doi: 10.3343/alm.2019.39.6.530 31240880 PMC6660342

[ref20] Hitch T. C. A. Riedel T. Oren A. Overmann J. Lawley T. D. Clavel T. (2021). Automated analysis of genomic sequences facilitates high-throughput and comprehensive description of bacteria. ISME Commun. 1:16. doi: 10.1038/s43705-021-00017-z, PMID: 36732617 PMC9723785

[ref21] Jain C. Rodriguez R. L. Phillippy A. M. Konstantinidis K. T. Aluru S. (2018). High throughput ANI analysis of 90K prokaryotic genomes reveals clear species boundaries. Nat. Commun. 9:5114. doi: 10.1038/s41467-018-07641-9, PMID: 30504855 PMC6269478

[ref22] Jiang L. Han H. L. (2023). *Cellulosimicrobium fucosivorans* is a later heterotypic synonym of *Cellulosimicrobium composti*. Int. J. Syst. Evol. Microbiol. 73:5848. doi: 10.1099/ijsem.0.005848, PMID: 37170960

[ref23] Kanehisa M. Sato Y. Kawashima M. Furumichi M. Tanabe M. (2016). KEGG as a reference resource for gene and protein annotation. Nucleic Acids Res. 44, D457–D462. doi: 10.1093/nar/gkv1070, PMID: 26476454 PMC4702792

[ref24] Karaoz U. Brodie E. L. (2022). microTrait: a toolset for a trait-based representation of microbial genomes. Front. Bioinform. 2:918853. doi: 10.3389/fbinf.2022.918853, PMID: 36304272 PMC9580909

[ref25] Karthik C. Kadirvelu K. Bruno B. Maharajan K. Rajkumar M. Manoj S. R.. (2021). *Cellulosimicrobium funkei* strain AR6 alleviate Cr(VI) toxicity in *Lycopersicon esculentum* by regulating the expression of growth responsible, stress tolerant and metal transporter genes. Rhizosphere 18:100351. doi: 10.1016/j.rhisph.2021.100351

[ref26] Krzywinski M. Schein J. Birol I. Connors J. Gascoyne R. Horsman D.. (2009). Circos: an information aesthetic for comparative genomics. Genome Res. 19, 1639–1645. doi: 10.1101/gr.092759.109 19541911 PMC2752132

[ref27] Lang-Yona N. Maier S. Macholdt D. S. Müller-Germann I. Yordanova P. Rodriguez-Caballero E.. (2018). Insights into microbial involvement in desert varnish formation retrieved from metagenomic analysis. Environ. Microbiol. Rep. 10, 264–271. doi: 10.1111/1758-2229.12634, PMID: 29488349

[ref28] Le Ho H. Tran-Van L. Quyen P. T. Q. Kim S. G. Jiang L. M. Chew K. W.. (2024). Bioinformatic approach to investigate larvae gut microbiota Cellulosimicrobium protaetiae via whole-genome analysis. Mol. Biotechnol. 1-9. doi: 10.1007/s12033-023-00984-9, PMID: 38231315

[ref29] Lee I. Ouk Kim Y. Park S. C. Chun J. (2016). OrthoANI: an improved algorithm and software for calculating average nucleotide identity. Int. J. Syst. Evol. Microbiol. 66, 1100–1103. doi: 10.1099/ijsem.0.000760, PMID: 26585518

[ref30] Letunic I. Bork P. (2021). Interactive tree of life (iTOL) v5: an online tool for phylogenetic tree display and annotation. Nucleic Acids Res. 49, W293–W296. doi: 10.1093/nar/gkab301, PMID: 33885785 PMC8265157

[ref1004] Li Z. (2009). Advances in marine microbial symbionts in the china sea and related pharmaceutical metabolites. Mar. Drugs. 7, 113–129. doi: 10.3390/md7020113, PMID: 19597576 PMC2707038

[ref31] Liu D. Zhang Y. Fan G. Sun D. Zhang X. Yu Z.. (2022). IPGA: a handy integrated prokaryotes genome and pan-genome analysis web service. iMeta 1:e55. doi: 10.1002/imt2.55, PMID: 38867900 PMC10989949

[ref32] Medlar A. J. Toronen P. Holm L. (2018). AAI-profiler: fast proteome-wide exploratory analysis reveals taxonomic identity, misclassification and contamination. Nucleic Acids Res. 46, W479–W485. doi: 10.1093/nar/gky359, PMID: 29762724 PMC6030964

[ref33] Meier-Kolthoff J. P. Auch A. F. Klenk H. P. Goker M. (2013). Genome sequence-based species delimitation with confidence intervals and improved distance functions. BMC Bioinformatics 14:60. doi: 10.1186/1471-2105-14-60, PMID: 23432962 PMC3665452

[ref34] Meier-Kolthoff J. P. Carbasse J. S. Peinado-Olarte R. L. Goker M. (2022). TYGS and LPSN: a database tandem for fast and reliable genome-based classification and nomenclature of prokaryotes. Nucleic Acids Res. 50, D801–D807. doi: 10.1093/nar/gkab902, PMID: 34634793 PMC8728197

[ref35] Meier-Kolthoff J. P. Klenk H. P. Goker M. (2014). Taxonomic use of DNA G+C content and DNA-DNA hybridization in the genomic age. Int. J. Syst. Evol. Microbiol. 64, 352–356. doi: 10.1099/ijs.0.056994-0, PMID: 24505073

[ref36] Monticelli J. Gerloni R. Farina C. Knezevich A. Dore F. Luzzati R. (2019). *Cellulosimicrobium cellulans* aortic prosthetic valve endocarditis. Access Microbiol. 1:e000068. doi: 10.1099/acmi.0.000068, PMID: 32974502 PMC7491936

[ref37] Nouioui I. Carro L. Garcia-Lopez M. Meier-Kolthoff J. P. Woyke T. Kyrpides N. C.. (2018). Genome-based taxonomic classification of the phylum Actinobacteria. Front. Microbiol. 9:2007. doi: 10.3389/fmicb.2018.02007, PMID: 30186281 PMC6113628

[ref38] Olson R. D. Assaf R. Brettin T. Conrad N. Cucinell C. Davis J. J.. (2023). Introducing the bacterial and viral bioinformatics resource center (BV-BRC): a resource combining PATRIC, IRD and ViPR. Nucleic Acids Res. 51, D678–D689. doi: 10.1093/nar/gkac1003, PMID: 36350631 PMC9825582

[ref39] Overbeek R. Olson R. Pusch G. D. Olsen G. J. Davis J. J. Disz T.. (2014). The SEED and the rapid annotation of microbial genomes using subsystems technology (RAST). Nucleic Acids Res. 42, D206–D214. doi: 10.1093/nar/gkt1226, PMID: 24293654 PMC3965101

[ref40] Parks D. H. Imelfort M. Skennerton C. T. Hugenholtz P. Tyson G. W. (2015). CheckM: assessing the quality of microbial genomes recovered from isolates, single cells, and metagenomes. Genome Res. 25, 1043–1055. doi: 10.1101/gr.186072.114 25977477 PMC4484387

[ref41] Prjibelski A. Antipov D. Meleshko D. Lapidus A. Korobeynikov A. (2020). Using SPAdes De Novo Assembler. Curr. Protoc. Bioinformatics 70:e102. doi: 10.1002/cpbi.102 32559359

[ref42] Rahi P. Muhle E. Scandola C. Touak G. Clermont D. (2024). Genome sequence-based identification of Enterobacter strains and description of Enterobacter pasteurii sp. nov. Microbiol. Spectr. 12:e0315023. doi: 10.1128/spectrum.03150-23, PMID: 38099614 PMC10783019

[ref43] Ren G. Yan Y. Nie Y. Lu A. Wu X. Li Y.. (2019). Natural extracellular electron transfer between semiconducting minerals and electroactive bacterial communities occurred on the rock varnish. Front. Microbiol. 10:293. doi: 10.3389/fmicb.2019.00293, PMID: 30886603 PMC6410676

[ref44] Richter M. Rossello-Mora R. Oliver Glockner F. Peplies J. (2016). JSpeciesWS: a web server for prokaryotic species circumscription based on pairwise genome comparison. Bioinformatics 32, 929–931. doi: 10.1093/bioinformatics/btv681, PMID: 26576653 PMC5939971

[ref45] Riesco R. Trujillo M. E. (2024). Update on the proposed minimal standards for the use of genome data for the taxonomy of prokaryotes. Int. J. Syst. Evol. Microbiol. 74:006300. doi: 10.1099/ijsem.0.006300, PMID: 38512750 PMC10963913

[ref46] Rivero M. Alonso J. Ramon M. F. Gonzales N. Pozo A. Marin I.. (2019). Infections due to Cellulosimicrobium species: case report and literature review. BMC Infect. Dis. 19:816. doi: 10.1186/s12879-019-4440-2, PMID: 31533642 PMC6751855

[ref47] Rodriguez-R L. M. Gunturu S. Harvey W. T. Rosselló-Mora R. Tiedje J. M. Cole J. R.. (2018). The microbial genomes atlas (MiGA) webserver: taxonomic and gene diversity analysis of Archaea and Bacteria at the whole genome level. Nucleic Acids Res. 46, W282–W288. doi: 10.1093/nar/gky467, PMID: 29905870 PMC6031002

[ref48] Rodriguez-R L. M. Konstantinidis K. T. (2016). The enveomics collection: a toolbox for specialized analyses of microbial genomes and metagenomes. PeerJ Preprints 4:e1900v1. doi: 10.7287/peerj.preprints.1900v1

[ref49] Rosconi F. Rudmann E. Li J. Surujon D. Anthony J. Frank M.. (2022). A bacterial pan-genome makes gene essentiality strain-dependent and evolvable. Nat. Microbiol. 7, 1580–1592. doi: 10.1038/s41564-022-01208-7, PMID: 36097170 PMC9519441

[ref50] Schomburg I. Chang A. Ebeling C. Gremse M. Heldt C. Huhn G.. (2004). BRENDA, the enzyme database: updates and major new developments. Nucleic Acids Res. 32, 431D–4433D. doi: 10.1093/nar/gkh081, PMID: 14681450 PMC308815

[ref51] Schumann P. Stackebrandt E. (2015). “Cellulosimicrobium,” in Bergey's manual of systematics of Archaea and Bacteria. ed. Whitman W. B. (Oxford, England: John Wiley & Sons, Ltd), 1–9.

[ref52] Schumann P. Weiss N. Stackebrandt E. (2001). Reclassification of *Cellulomonas cellulans* (Stackebrandt and Keddie 1986) as *Cellulosimicrobium cellulans* gen. nov., comb. nov. Int. J. Syst. Evol. Microbiol. 51, 1007–1010. doi: 10.1099/00207713-51-3-1007, PMID: 11411667

[ref53] Schwengers O. Hoek A. Fritzenwanker M. Falgenhauer L. Hain T. Chakraborty T.. (2020). ASA3P: an automatic and scalable pipeline for the assembly, annotation and higher-level analysis of closely related bacterial isolates. PLoS Comput. Biol. 16:e1007134. doi: 10.1371/journal.pcbi.1007134 32134915 PMC7077848

[ref54] Sharpton T. J. Stajich J. E. Rounsley S. D. Gardner M. J. Wortman J. R. Jordar V. S.. (2009). Comparative genomic analyses of the human fungal pathogens Coccidioides and their relatives. Genome Res. 19, 1722–1731. doi: 10.1101/gr.087551.108, PMID: 19717792 PMC2765278

[ref55] Shu W. S. Huang L. N. (2022). Microbial diversity in extreme environments. Nat. Rev. Microbiol. 20, 219–235. doi: 10.1038/s41579-021-00648-y 34754082

[ref56] Sims D. B. Hudson A. C. Keller J. E. Strange M. Buch A. C. Ferrari D.. (2022). Trace elements migrating from tailings to rock varnish laminated sediments in an old mining region from Nelson, Nevada, USA. Int. J. Sediment Res. 37, 202–213. doi: 10.1016/j.ijsrc.2021.08.001

[ref57] Sultanpuram V. R. Mothe T. Chintalapati S. Chintalapati V. R. (2015). Cellulosimicrobium aquatile sp. nov., isolated from Panagal reservoir, Nalgonda, India. Antonie Van Leeuwenhoek 108, 1357–1364. doi: 10.1007/s10482-015-0588-y, PMID: 26373417

[ref58] Sun J. Lu F. Luo Y. Bie L. Xu L. Wang Y. (2023). OrthoVenn3: an integrated platform for exploring and visualizing orthologous data across genomes. Nucleic Acids Res. 51, W397–W403. doi: 10.1093/nar/gkad313, PMID: 37114999 PMC10320085

[ref59] Tatusova T. DiCuccio M. Badretdin A. Chetvernin V. Nawrocki E. P. Zaslavsky L.. (2016). NCBI prokaryotic genome annotation pipeline. Nucleic Acids Res. 44, 6614–6624. doi: 10.1093/nar/gkw569, PMID: 27342282 PMC5001611

[ref60] Wirbel J. Bhatt A. S. Probst A. J. (2024). The journey to understand previously unknown microbial genes. Nature 626, 267–269. doi: 10.1038/d41586-024-00077-w 38291331

[ref61] Wu W. Feng Y. Zong Z. (2020). Precise species identification for Enterobacter: a genome sequence-based study with reporting of two novel species, Enterobacter quasiroggenkampii sp. nov. and Enterobacter quasimori sp. nov. mSystems 5, 5:e00527-20. doi: 10.1128/mSystems.00527-20, PMID: 32753511 PMC7406230

[ref1005] Yoon J.-H. Kang S.-J. Schumann P. Oh T.-K. (2007). Cellulosimicrobium terreum sp. nov., isolated from soil. Int J Syst Evol Microbiol. 57, 2493–2497. doi: 10.1099/ijs.0.64889-0, PMID: 17978207

[ref62] Yoon S. H. Ha S. M. Kwon S. Lim J. Kim Y. Seo H.. (2017). Introducing EzBioCloud: a taxonomically united database of 16S rRNA gene sequences and whole-genome assemblies. Int. J. Syst. Evol. Microbiol. 67, 1613–1617. doi: 10.1099/ijsem.0.001755, PMID: 28005526 PMC5563544

