# Supplementary material for: Comparative genomic characterization of Cellulosimicrobium funkei isolate RVMD1 from Ma’an desert rock varnish challenges Cellulosimicrobium systematics
Source: Front Microbiol. 2024 Nov 7;15:1445943. doi: 10.3389/fmicb.2024.1445943 (PMC11579708; doi:10.3389/fmicb.2024.1445943)
Supplement: Supplementary file 1 [file Supplementary_file_1.docx]

Supplementary Material

**
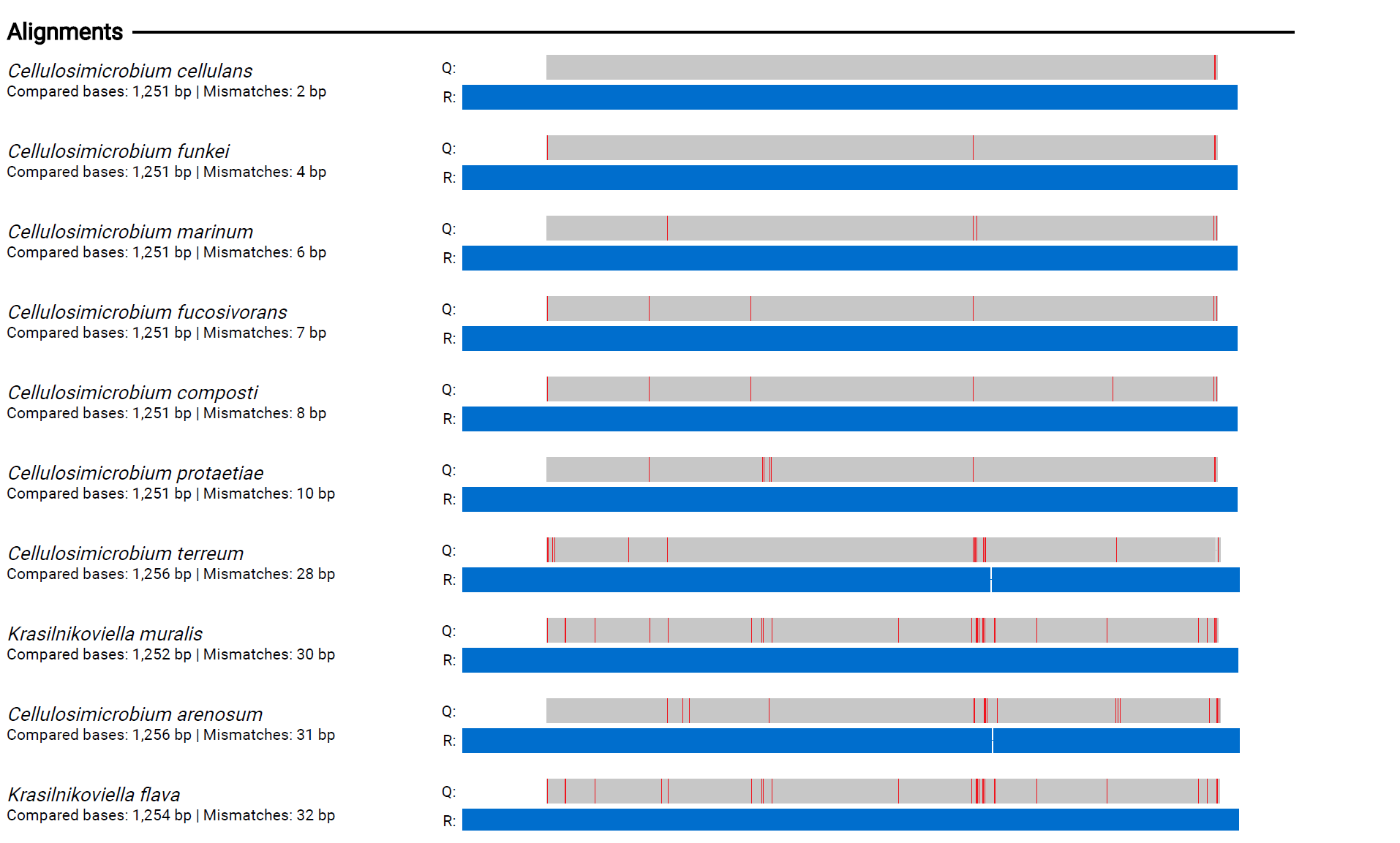
**

**Fig S1:** **Alignment visualization of top hits for bacteria isolate RVMD1 through 16S rRNA gene (accession number :**[**OR570906**](https://www.ncbi.nlm.nih.gov/nuccore/OR570906)**) sequencing analysis in EzBioCloud Pro 16S-ID app (Yoon et al., 2017).** The alignments are generated using the TrueBac2 analysis engine (version 0.1) with the TB-Genome-BA database (version 221108).


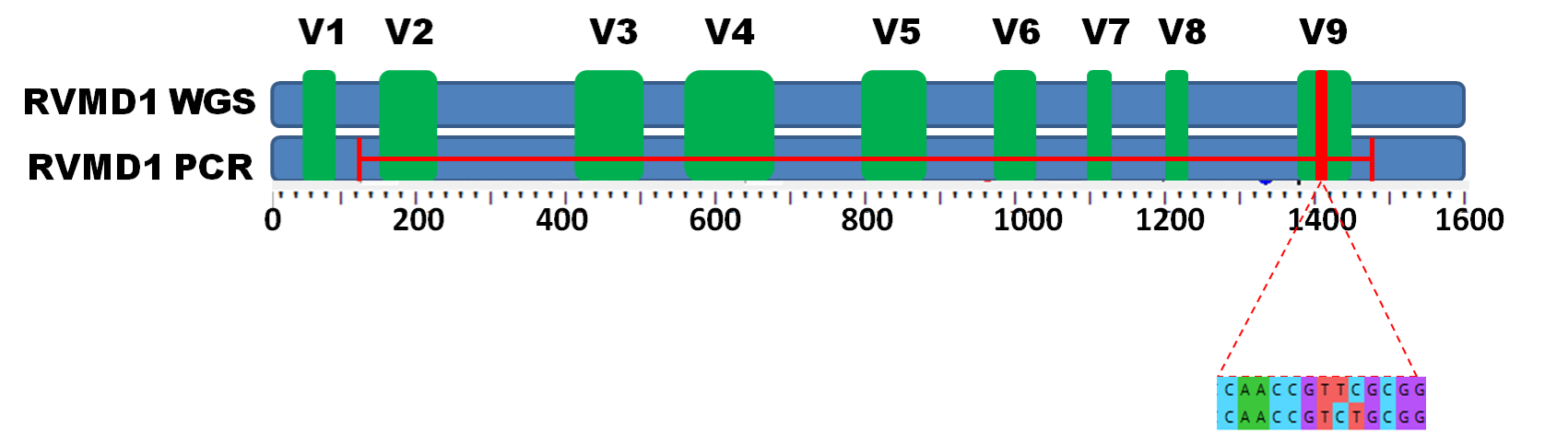


**Fig S2:** Sequence comparison of RVMD1 isolate between Sanger (1,251 bp) and WGS (1,467 bp) showing two nucleotide substitutions (T ↔ C and C ↔ T) in the V9 region across the 16S rRNA gene.

**Table S1: Detailed identification of taxa closely related to *C. funkei* RVMD1 isolate comprehensive analysis of 16S rRNA, *recA*, *rplC* genes extracted from WG and Mash identity using the TrueBac ID-Genome system (Ha et al., 2019)**

| Rank | Hit Taxon | 16S Similarity(%) | *recA* Identity(%) | *rplC* Identity(%) | Mash Identity(%) |
| --- | --- | --- | --- | --- | --- |
| 1 | *C. funkei* | 99.79 | 99.012 | 99.849 | 97.81 |
| 2 | *C. terreum* | 97.50 | 99.012 | 99.849 | 97.81 |
| 3 | *C. cellulans* | 100.00 | 97.125 | 99.397 | 96.08 |
| 6 | *Luteimicrobium xylanilyticum* | 98.96 | 92.429 | N/A | N/A |
| 8 | *Isoptericola dokdonensis* | 97.36 | 89.362 | N/A | N/A |
| 14 | *Xylanimonas cellulosilytica* | 97.09 | 88.536 | N/A | N/A |
| 9 | *Isoptericola jiangsuensis* | 96.75 | 75.714 | N/A | N/A |
| 4 | *C. marinum* | 95.77 | N/A | N/A | N/A |
| 5 | *C. arenosum* | 95.70 | N/A | N/A | N/A |
| 7 | *Krasilnikoviella flava* | 95.35 | N/A | 90.205 | N/A |
| 10 | *Oerskovia turbata* | 95.28 | N/A | 90.799 | 84.34 |
| 11 | *Oerskovia enterophila* | 95.08 | N/A | N/A | 84.88 |
| 12 | *Promicromonospora kroppenstedtii* | 94.85 | N/A | 90.205 | N/A |
| 13 | *Antribacter gilvus* | 94.73 | N/A | N/A | N/A |
| 15 | *Cellulomonas iranensis* | 94.38 | N/A | 90.199 | N/A |
| 16 | *Cellulomonas telluris* | 94.38 | N/A | 90.505 | N/A |


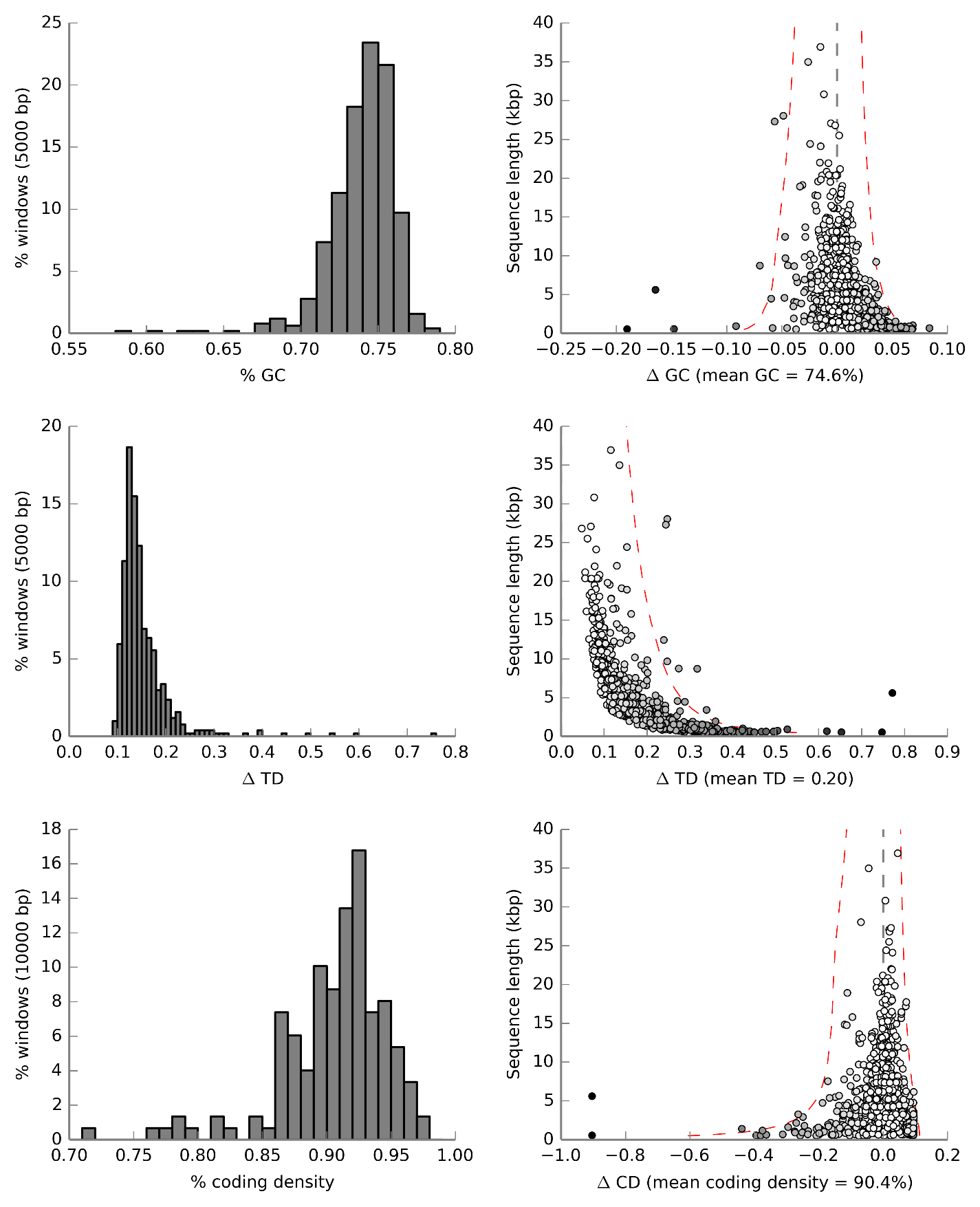


**Fig S3: Genomic feature distributions in *C. funkei* RVMD1 Using CheckM v1.0.18 in KBase Platform (Parks et al., 2015). (A)** GC content across 5,000 bp windows peaks within a stable range, (B) Scatter plot reveals tetranucleotide signature deviations, with outliers indicated by red dashed lines. (C) Merges the coding density histogram and deviation scatter plot, depicting a uniform gene distribution with some sequences deviating from the mean, indicative of genomic homogeneity with occasional anomalies.

**
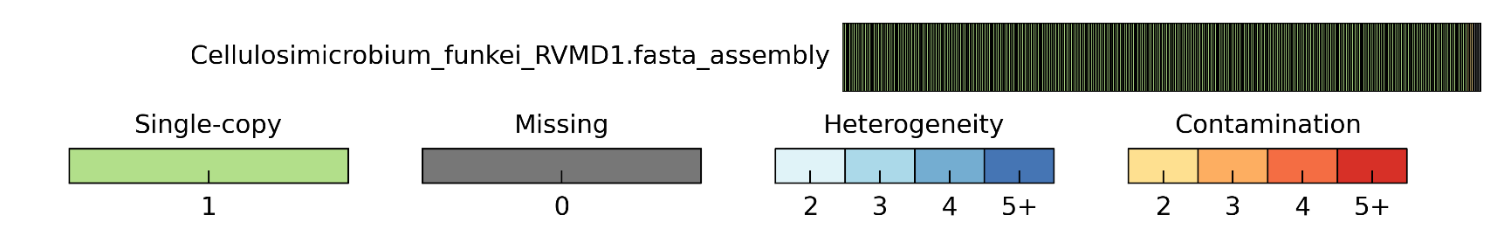
**

**Fig S4: CheckM assessment of *C. funkei* RVMD1 genome completeness and contamination.** A full set of single-copy genes is shown by the green bar, denoting genome completeness. The grey bar confirms no missing genes, indicating a comprehensive genome assembly. Blue bars represent low genome heterogeneity, suggesting consistency, and the yellow-to-red gradient bars mark the potential contamination levels, with darker reds signaling higher concern, overall pointing to a well-assembled genome with minimal contamination.

| Virulence Factor Category | Gene IDs and VF Gene Names | VF Factor IDs |
| --- | --- | --- |
| Immune Modulation/Inflammatory Signaling | NP_540330 (*gmd*), NP_878992 (*bplD*) | VFG002225, VFG038348 |
| Effector Delivery System (Type III SS) | YP_001144298 (a*scN*), YP_111547 (*bsaS*), NP_880888 (*bscN*), NP_250388 (*pscN*) | VFG048478, VFG048468, VFG038532, VFG002459 |
| Nutritional/Metabolic Factor (Iron Uptake) | YP_002918372.1 (*fepG*), YP_002918373.1 (*fepD*), YP_002918371.1 (*fepC*) | VFG001259, VFG000048, VFG001386 |
| Adherence (Type IV Pili) | YP_855994 (*flpF*) | VFG000035 |
| Motility (Flagella Assembly) | NP_250137 (*fliP*), YP_106655 (*fliP*), YP_855907 (*fliI*), YP_106853 *(fliK),* NP_249795 (*fliI*), YP_106855 (*fliI*), YP_109899 (c*heR*) | VFG037028, VFG002494, VFG038638, VFG001386, VFG048488 |
| Regulation | NP_215271 (*phoP*), NP_217099 (*relA*), NP_217227 *(ideR)* | VFG001826, VFG002497, VFG001254, VFG002499, VFG002528 |
| Stress Survival | NP_273273 (*katA*) | VFG000192 |
| Effector Delivery System (Type VI SS) | NP_248764 (*ppkA*) | VFG002060 |
| Nutritional/Metabolic Factor (Carbohydrate Uptake) | YP_177728 (*icl*) | VFG001406, VFG001381 |

**Table S2**: Classification of virulence factor genes in *C. funkei* RVMD1.This table lists virulence factor (VF) genes in *C. funkei* RVMD1, identified using TrueBacTM and VFDB. It categorizes genes by virulence roles and links each to its VFDB ID. Identity percentages range from 64.67% to 74.96%, and query coverage percentages vary from 51.74% to 88.01%, reflecting gene similarity and alignment with known VF sequences in VFDB.

**Table S3:** Antimicrobial Resistance (AMR) genes in *C. funkei* RVMD1 were identified using the Genome Annotation Service in PATRIC (BV-BRC), which employs a k-mer-based detection method.

| AMR Mechanism | Genes |
| --- | --- |
| Antibiotic target in susceptible species | *Alr, Ddl, dxr, EF-G, EF-Tu, folA, Dfr, folP, gyrA, gyrB, inhA, fabI, Iso-tRNA, kasA, MurA, rho, rpoB, rpoC, S10p, S12p* |
| Antibiotic target replacement protein | *FabG, HtdX* |
| Gene conferring resistance via absence | *gidB* |
| Protein altering cell wall charge conferring antibiotic resistance | *GdpD, PgsA* |
| Regulator modulating expression of antibiotic resistance genes | *MtrA, MtrB* |

**Table S4:** A TYGS-based analysis of 43 genomes encompassing the *Cellulosimicrobium* genus and *C. funkei* RVMD1, highlighting base pairs, G+C content, protein counts, species cluster, CheckM completeness, and CheckM contamination.

| No | Species cluster | Preferred name | Base pairs | Percent G+C | No. proteins | CheckM Completeness | CheckM Contamination |
| --- | --- | --- | --- | --- | --- | --- | --- |
| 1 | 1 | *Cellulosimicrobium* sp. XJ-DQ-B-000 | 3,043,158 | 74.57 | 2723 | 75.36 | 1.43 |
| 2 | 1 | *C. cellulans* strain NEB113 | 4,219,812 | 74.64 | 3704 | 98.19 | 1.69 |
| 3 | 1 | *C. cellulans* strain NBRC 103059 | 4,212,504 | 74.67 | 3704 | 98.19 | 1.69 |
| 4 | 1 | *C. cellulans* strain ATCC 21606 | 4,209,480 | 74.68 | 3711 | 98.19 | 1.69 |
| 5 | 1 | *Cellulosimicrobium* sp. 72-3 | 4,220,771 | 74.62 | 3719 | 98.28 | 1.69 |
| 6 | 1 | *Cellulosimicrobium* sp. TH-20 | 4,265,546 | 74.60 | 3770 | 97.88 | 1.70 |
| 7 | 1 | *C. funkei* strain P112 | 4,266,893 | 74.71 | 3791 | 98.62 | 2.00 |
| 8 | 1 | *C. aquatile* strain 3bp | 4,317,712 | 74.73 | 3793 | 98.12 | 1.40 |
| 9 | 1 | Uncultured *C.*  sp. isolate SRR6216767 MAG genomic | 4,170,669 | 74.75 | 3799 | 96.84 | 1.76 |
| 10 | 1 | *Cellulosimicrobium sp.* TH-20 strain DE0020 | 4,327,705 | 74.57 | 3833 | 98.14 | 1.44 |
| 11 | 1 | *Cellulosimicrobium* sp. SL-1 | 4,330,690 | 74.69 | 3850 | 98.45 | 1.78 |
| 12 | 1 | *Cellulosimicrobium* sp. KWT-B | 4,412,091 | 74.61 | 3851 | 98.99 | 2.14 |
| 13 | 1 | *Cellulosimicrobium* sp. JZ28 | 4,378,193 | 74.69 | 3898 | 95.44 | 1.90 |
| 14 | 1 | *Cellulosimicrobium* sp. TH-20 strain DE0282 | 4,346,475 | 74.56 | 3926 | 98.28 | 1.78 |
| 15 | 1 | *C. funkei* strain JCM 14302 | 4,377,803 | 74.54 | 3930 | 98.39 | 1.91 |
| 16 | 1 | *C. aquatile* strain WB02 D5 03 | 4,403,989 | 74.66 | 3955 | 98.83 | 1.73 |
| 17 | 1 | *C. cellulans* strain ORNL-0100 | 4,411,608 | 74.43 | 3957 | 97.79 | 2.18 |
| 18 | 1 | *C. funkei* NBRC 104118 | 4,370,484 | 74.55 | 3962 | 98.19 | 1.91 |
| 19 | 1 | *Cellulosimicrobium* sp. MM | 3,846,862 | 74.45 | 4153 | 60.28 | 10.14 |
| 20 | 1 | *C. cellulans* LMG 16121 | 4,276,035 | 74.30 | 4183 | 97.88 | 2.23 |
| 21 | 1 | *C. cellulans* strain 1C2-5 | 3,834,263 | 73.99 | 4232 | 93.85 | 1.76 |
| 22 | 1 | *C. cellulans* strain 1B1-1 | 4,368,117 | 74.52 | 4248 | 97.99 | 2.27 |
| 23 | 1 | ***C. funkei* RVMD1** | **4,264,015** | **74.59** | **4449** | **98.55** | **0.87** |
| 24 | 2 | *C. arenosum* strain KCTC 49039 | 3,951,297 | 72.88 | 3431 | 96.31 | 3.82 |
| 25 | 3 | *C. cellulans* strain 2020WEIHUA | 4,661,058 | 73.87 | 4054 | 96.63 | 2.96 |
| 26 | 4 | *C. cellulans* strain DE0111 | 4,576,205 | 75.57 | 4143 | 91.79 | 7.44 |
| 27 | 5 | *C. cellulans* strain JZ5 | 3,685,650 | 74.30 | 3594 | 95.73 | 2.17 |
| 28 | 6 | *C. cellulans* strain MP1 | 4,580,223 | 73.92 | 4065 | 98.63 | 1.91 |
| 29 | 7 | *C. cellulans* strain NBRC 15516 | 4,595,203 | 73.97 | 4046 | 97.75 | 2.70 |
| 30 | 7 | *C. cellulans* strain ZKA17 | 4,721,213 | 73.84 | 4107 | 98.72 | 2.30 |
| 31 | 8 | *C. cellulans* strain DSM 20106 | 4,788,922 | 74.14 | 4094 | 98.76 | 2.66 |
| 32 | 8 | *C. cellulans* strain PSBB019 | 4,799,856 | 74.14 | 4199 | 96.34 | 2.77 |
| 33 | 9 | *C. cellulans* strain PW | 4,672,389 | 74.27 | 4034 | 98.19 | 2.23 |
| 34 | 10 | *C. composti* strain SE3 | 4,307,178 | 74.79 | 3793 | 98.77 | 0.65 |
| 35 | 10 | *C. composti* strain BIT-GX5 | 4,316,226 | 74.57 | 3892 | 98.60 | 0.75 |
| 36 | 11 | *C. marinum* strain NBRC 110994 | 3,830,619 | 74.31 | 3378 | 97.46 | 3.15 |
| 37 | 12 | *C. protaetiae* strain BI34 | 4,784,181 | 73.89 | 4097 | 98.23 | 1.72 |
| 38 | 13 | *Cellulosimicrobium* sp. CUA-896 | 3,727,087 | 74.64 | 3875 | 64.86 | 1.42 |
| 39 | 14 | *C. cellulans* isolate CTOTU50488 | 4,588,304 | 74.18 | 4041 | 98.32 | 2.87 |
| 40 | 14 | *C. cellulans* F16 | 4,602,720 | 74.21 | 4050 | 97.57 | 2.87 |
| 41 | 14 | *C. funkei* strain U11 | 4,658,729 | 74.20 | 4141 | 97.87 | 2.47 |
| 42 | 14 | *Cellulosimicrobium* sp. I38E | 4,709,106 | 74.18 | 4180 | 98.41 | 2.83 |
| 43 | 15 | *Cellulosimicrobium* sp. Marseille-Q4280 | 4,860,837 | 73.68 | 4309 | 98.06 | 3.79 |
| 44 | 16 | *Cellulosimicrobium* sp. SH8 | 4,727,140 | 74.13 | 4133 | 98.40 | 2.45 |


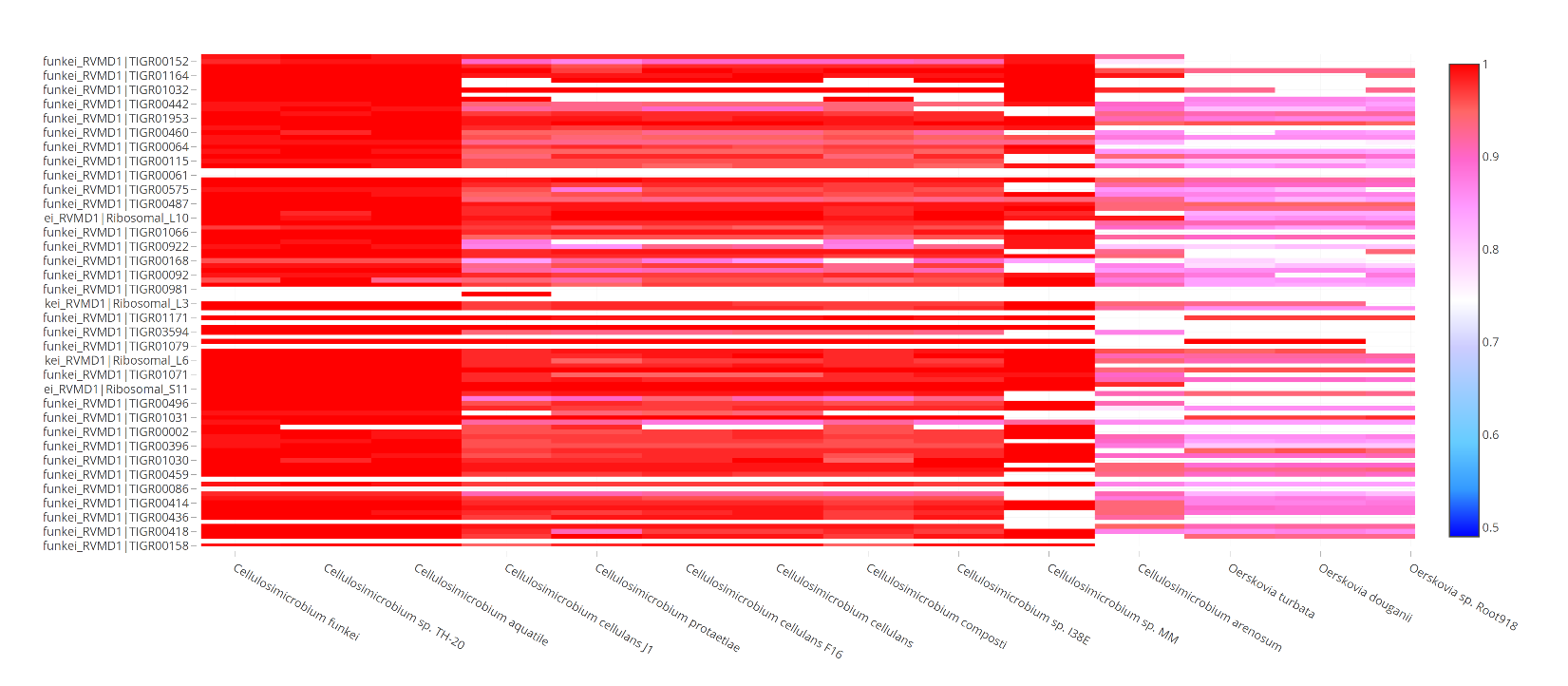


**Fig S5:** Similarity matrix Heatmap of essential genes from *C. funkei* RVMD1. Essential genes were extracted by the Microbial Genomes Atlas (MiGA) webserver (Rodriguez-R et al., 2018), and their proteomic similarities to the top related species were visualized using the AAI-profiler web server (Medlar et al., 2018).


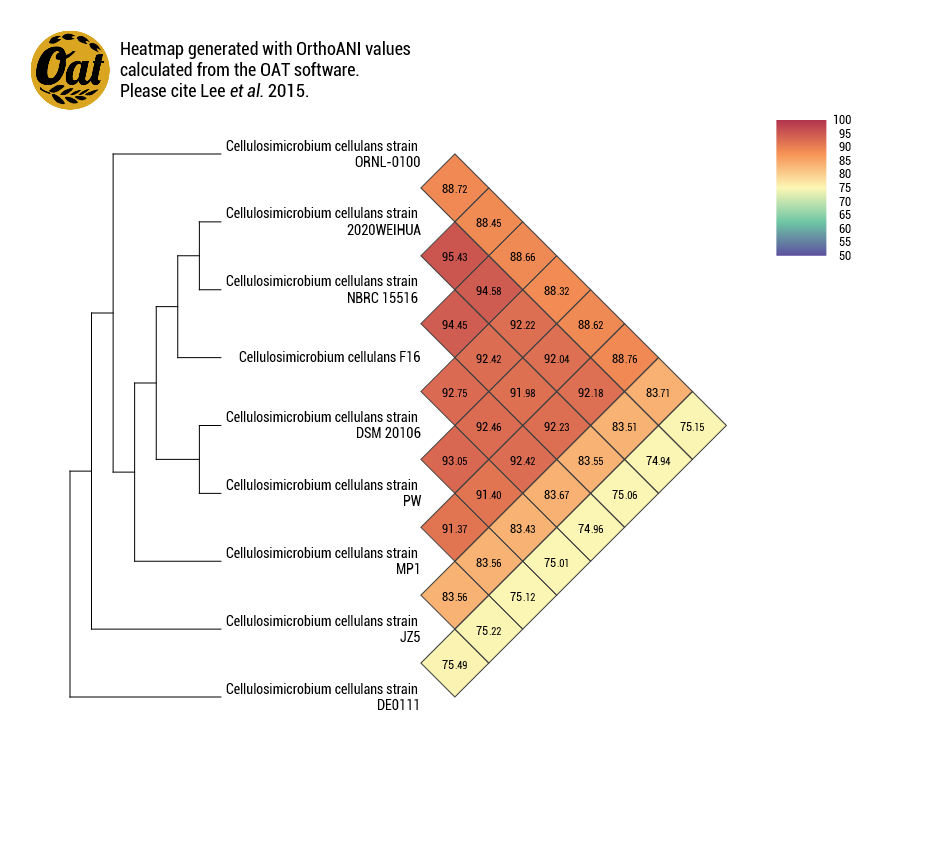


**Fig S6**: Heatmap of all-vs-all Orthologous Average Nucleotide Identity (OrthoANI) among selected representative *C. cellulans* species from different clusters. The ANI phylogenetic tree was constructed based on OrthoANI values calculated from the OAT software.

**Table S5:** All-vs-all ANIb values of all *C. cellulans* species from different species clusters

**Table S6:** Comparison of CAZyme families (GH, GT, PL, CE, AA, and CBM) in *C. funkei* RVMD1 and other species in the *C. cellulans* group, analyzed via Protologger (Hitch et al., 2021).

| Strain | ZKA17 | CTOTU50488 | F16 | PW | PSBB019 | ORNL-0100 | NEB113 | NBRC 103059 | NBRC 15516 | MP1 | JZ5 | DSM 20106 | DE0111 | ATCC 21606 | 2020WEIHUA | 1C2-5 | 1B1-1 | LMG 16121 |
| --- | --- | --- | --- | --- | --- | --- | --- | --- | --- | --- | --- | --- | --- | --- | --- | --- | --- | --- |
| ZKA17 | 100 | 93.94 | 93.94 | 91.42 | 91.98 | 87.85 | 87.95 | 87.96 | 96.8 | 91.63 | 82.49 | 92.02 | 74.43 | 87.96 | 95.5 | 88.38 | 88.12 | 88 |
| CTOTU50488 | 93.78 | 100 | 98.65 | 92.06 | 92.46 | 88.1 | 88.27 | 88.26 | 93.97 | 91.98 | 82.66 | 92.41 | 74.52 | 88.26 | 94.08 | 88.84 | 88.46 | 88.36 |
| F16 | 93.87 | 98.68 | 100 | 92.01 | 92.46 | 88.14 | 88.26 | 88.26 | 94.03 | 91.97 | 82.8 | 92.51 | 74.52 | 88.26 | 94.08 | 88.81 | 88.44 | 88.44 |
| PW | 91.34 | 92.06 | 92.06 | 100 | 92.78 | 87.96 | 88.07 | 88.08 | 91.48 | 90.81 | 82.66 | 92.76 | 74.77 | 88.07 | 91.48 | 88.56 | 88.29 | 88.14 |
| PSBB019 | 91.7 | 92.18 | 92.25 | 92.59 | 100 | 87.45 | 87.61 | 87.59 | 91.91 | 90.56 | 82.32 | 99.03 | 74.63 | 87.61 | 91.66 | 88.14 | 87.91 | 87.64 |
| ORNL-0100 | 88.5 | 88.66 | 88.71 | 88.56 | 88.32 | 100 | 97.42 | 97.42 | 88.48 | 88.62 | 83.22 | 88.34 | 75.24 | 97.42 | 88.62 | 97.27 | 96.72 | 97.01 |
| NEB113 | 88.65 | 88.89 | 88.98 | 88.72 | 88.47 | 97.51 | 100 | 100 | 88.72 | 88.92 | 83.28 | 88.47 | 75.22 | 100 | 88.67 | 97.73 | 96.93 | 98.23 |
| NBRC 103059 | 88.69 | 88.94 | 88.98 | 88.68 | 88.43 | 97.53 | 100 | 100 | 88.71 | 88.99 | 83.31 | 88.45 | 75.23 | 100 | 88.68 | 97.77 | 96.93 | 98.29 |
| NBRC 15516 | 96.86 | 94.06 | 94.04 | 91.55 | 92.13 | 87.92 | 88.12 | 88.11 | 100 | 91.65 | 82.61 | 92.1 | 74.62 | 88.12 | 95.18 | 88.63 | 88.23 | 88.24 |
| MP1 | 91.6 | 92.18 | 92.18 | 91.01 | 91.01 | 88.31 | 88.47 | 88.47 | 91.67 | 100 | 82.72 | 91.03 | 74.76 | 88.47 | 91.7 | 89.01 | 88.49 | 88.57 |
| JZ5 | 83.12 | 83.21 | 83.25 | 83.19 | 83.1 | 83.27 | 83.21 | 83.2 | 83.18 | 83.13 | 100 | 83.09 | 75.41 | 83.19 | 83.14 | 83.87 | 83.22 | 83.34 |
| DSM 20106 | 91.7 | 92.11 | 92.25 | 92.52 | 99.03 | 87.57 | 87.63 | 87.62 | 91.76 | 90.6 | 82.22 | 100 | 74.54 | 87.63 | 91.62 | 88.21 | 88 | 87.7 |
| DE0111 | 74.76 | 74.84 | 74.95 | 74.95 | 74.87 | 75.03 | 75.12 | 75.11 | 74.78 | 74.91 | 75.08 | 74.83 | 100 | 75.12 | 74.72 | 75.48 | 75.1 | 75.12 |
| ATCC 21606 | 88.65 | 88.86 | 88.88 | 88.63 | 88.39 | 97.51 | 100 | 100 | 88.72 | 88.93 | 83.34 | 88.38 | 75.27 | 100 | 88.67 | 97.77 | 96.86 | 98.24 |
| 2020WEIHUA | 95.51 | 94.08 | 94.09 | 91.47 | 91.97 | 88.02 | 88.05 | 88.05 | 95.18 | 91.58 | 82.51 | 91.9 | 74.42 | 88.04 | 100 | 88.57 | 88.24 | 88.22 |
| 1C2-5 | 88.72 | 89.07 | 89.1 | 88.81 | 88.61 | 97.42 | 97.79 | 97.79 | 88.84 | 88.93 | 83.38 | 88.65 | 75.21 | 97.79 | 88.84 | 100 | 97.11 | 97.72 |
| 1B1-1 | 88.4 | 88.67 | 88.58 | 88.52 | 88.37 | 96.59 | 96.64 | 96.63 | 88.39 | 88.62 | 82.93 | 88.34 | 75.03 | 96.63 | 88.43 | 96.87 | 100 | 96.44 |
| LMG 16121 | 88.45 | 88.69 | 88.73 | 88.4 | 88.25 | 97.06 | 98.14 | 98.14 | 88.5 | 88.77 | 83.18 | 88.23 | 75.06 | 98.14 | 88.57 | 97.67 | 96.59 | 100 |

| **Category** | **RVMD1 (Total: 288 CAZymes)** | **ORNL-0100 (Total: 274 CAZymes)** | **JCM 14302 (Total: 277 CAZymes)** |
| --- | --- | --- | --- |
| **Glycoside Hydrolase (GH) Families** | GH16: 5, GH15: 1, GH13: 21, GH18: 5, GH19: 5, GH74: 1, GH39: 1, GH51: 2, GH71: 1, GH30: 1, GH31: 1, GH32: 2, GH33: 2, GH36: 2, GH146: 2, GH127: 1, GH101: 2, GH121: 1, GH25: 2, GH105: 3, GH77: 1, GH65: 3, GH64: 1, GH23: 9, GH20: 2, GH43: 8, GH42: 1, GH4: 1, GH6: 2, GH0: 7, GH1: 17, GH2: 1, GH3: 7, GH28: 1, GH130: 6, GH114: 1, GH94: 2, GH92: 3 | GH15: 1, GH94: 2, GH20: 3, GH23: 6, GH105: 6, GH13: 24, GH19: 5, GH130: 3, GH1: 13, GH0: 6, GH16: 4, GH31: 1, GH65: 2, GH3: 4, GH92: 3, GH74: 1, GH28: 1, GH33: 3, GH77: 1, GH101: 1, GH18: 5, GH43: 6, GH146: 1, GH51: 1, GH36: 1, GH42: 1, GH30: 1, GH6: 2, GH64: 1, GH32: 1, GH4: 1, GH2: 1, GH114: 1 | GH92: 5, GH130: 5, GH0: 6, GH1: 13, GH94: 2, GH3: 5, GH16: 4, GH13: 24, GH87: 1, GH105: 8, GH4: 2, GH114: 1, GH33: 3, GH18: 5, GH77: 1, GH19: 5, GH65: 2, GH43: 6, GH74: 1, GH28: 1, GH2: 1, GH32: 1, GH6: 2, GH64: 1, GH101: 1, GH23: 6, GH31: 1, GH73: 1, GH20: 3, GH146: 1, GH51: 1, GH36: 1, GH42: 1, GH30: 1, GH15: 1 |
| **Glycosyl Transferase (GT) Families** | GT26: 2, GT35: 1, GT30: 1, GT32: 1, GT13: 1, GT20: 1, GT39: 1, GT28: 1, GT0: 5, GT1: 4, GT2: 34, GT4: 27, GT5: 1, GT51: 4, GT9: 1 | GT4: 22, GT2: 32, GT20: 1, GT26: 1, GT0: 6, GT39: 1, GT94: 1, GT35: 1, GT30: 1, GT28: 4, GT32: 1, GT1: 5, GT51: 3, GT5: 1 | GT28: 4, GT51: 3, GT1: 5, GT32: 1, GT2: 31, GT4: 25, GT9: 3, GT26: 2, GT30: 1, GT0: 1, GT35: 1, GT5: 1, GT20: 1, GT39: 1, GT94: 1 |
| **Polysaccharide Lyase (PL) Families** | - | PL0: 1 | PL0: 1 |
| **Carbohydrate Esterase (CE) Families** | CE12: 6, CE14: 9, CE7: 1, CE4: 4, CE1: 2, CE0: 1, CE9: 1 | CE7: 1, CE4: 3, CE14: 6, CE9: 2, CE12: 4, CE1: 3, CE0: 2 | CE12: 4, CE0: 2, CE14: 5, CE1: 3, CE4: 3, CE9: 2, CE7: 1 |
| **Auxiliary Activity (AA) Families** | AA10: 1 | AA10: 1 | - |
| **Carbohydrate-Binding Module (CBM) Families** | CBM6: 7, CBM50: 16, CBM12: 1, CBM13: 7, CBM16: 1, CBM48: 9, CBM32: 3, CBM2: 5, CBM35: 3, CBM22: 2, CBM20: 1, CBM38: 2, CBM25: 1, CBM5: 14 | CBM32: 3, CBM13: 10, CBM50: 22, CBM2: 6, CBM20: 1, CBM25: 1, CBM51: 1, CBM5: 13, CBM48: 11, CBM6: 7, CBM38: 4, CBM16: 1, CBM22: 2, CBM12: 2, CBM4: 1, CBM35: 2 | CBM2: 5, CBM48: 11, CBM50: 18, CBM6: 7, CBM35: 2, CBM13: 8, CBM32: 4, CBM38: 4, CBM5: 13, CBM12: 2, CBM4: 1, CBM16: 1, CBM22: 2, CBM20: 1, CBM25: 1, CBM51: 1 |


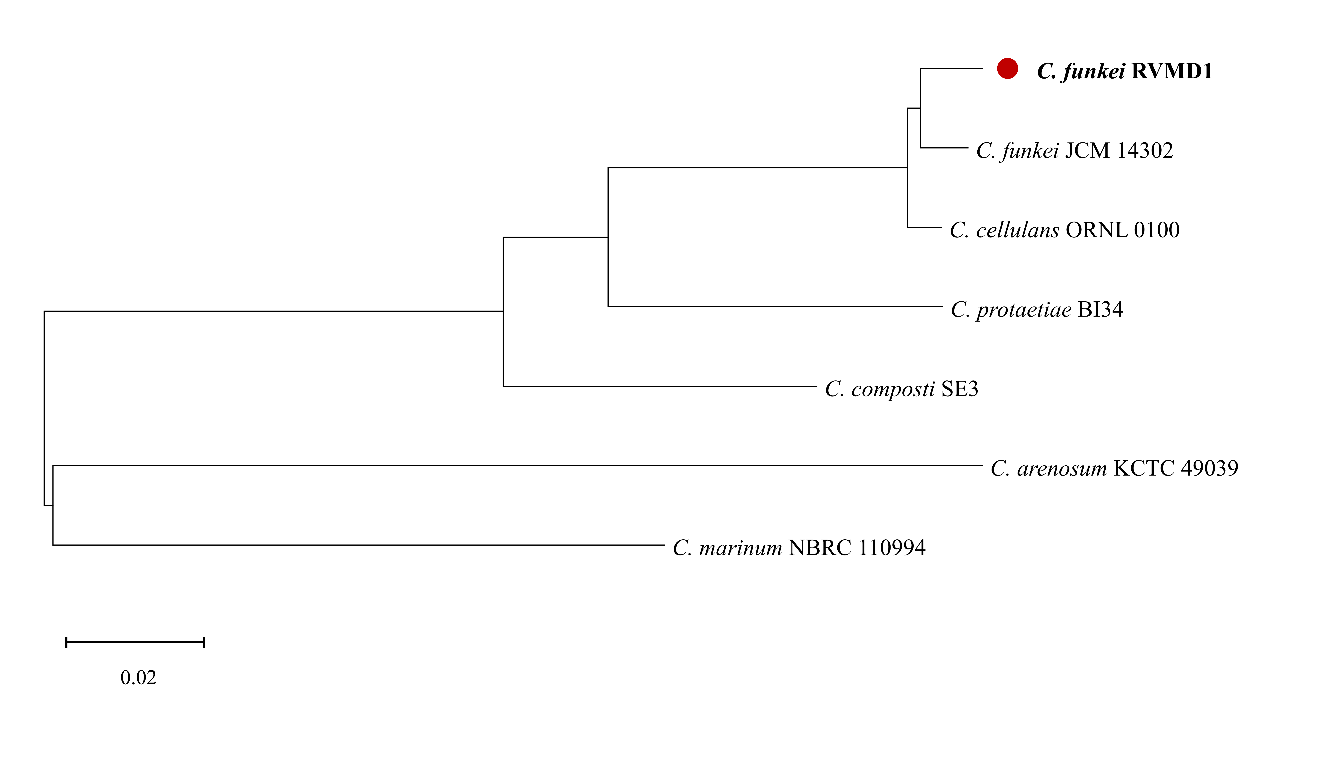


**Fig S7**: Phylogenomic Analysis Using Core Genomes: Utilizing 1,434 single-copy shared genes encoding 470,005 amino acids, a phylogenetic tree was constructed to illustrate evolutionary relationships. The analysis, based on a comprehensive dataset of 470,005 positions, was performed with MEGA11 software (Tamura et al., 2021). Gene clusters were identified through OrthoVenn3 (Sun et al., 2023). This process involved the extraction of highly conserved single-copy genes, concatenated alignment via Muscle, sequence trimming with Trimal, and phylogenetic inference through FastTree using the maximum likelihood method.

**
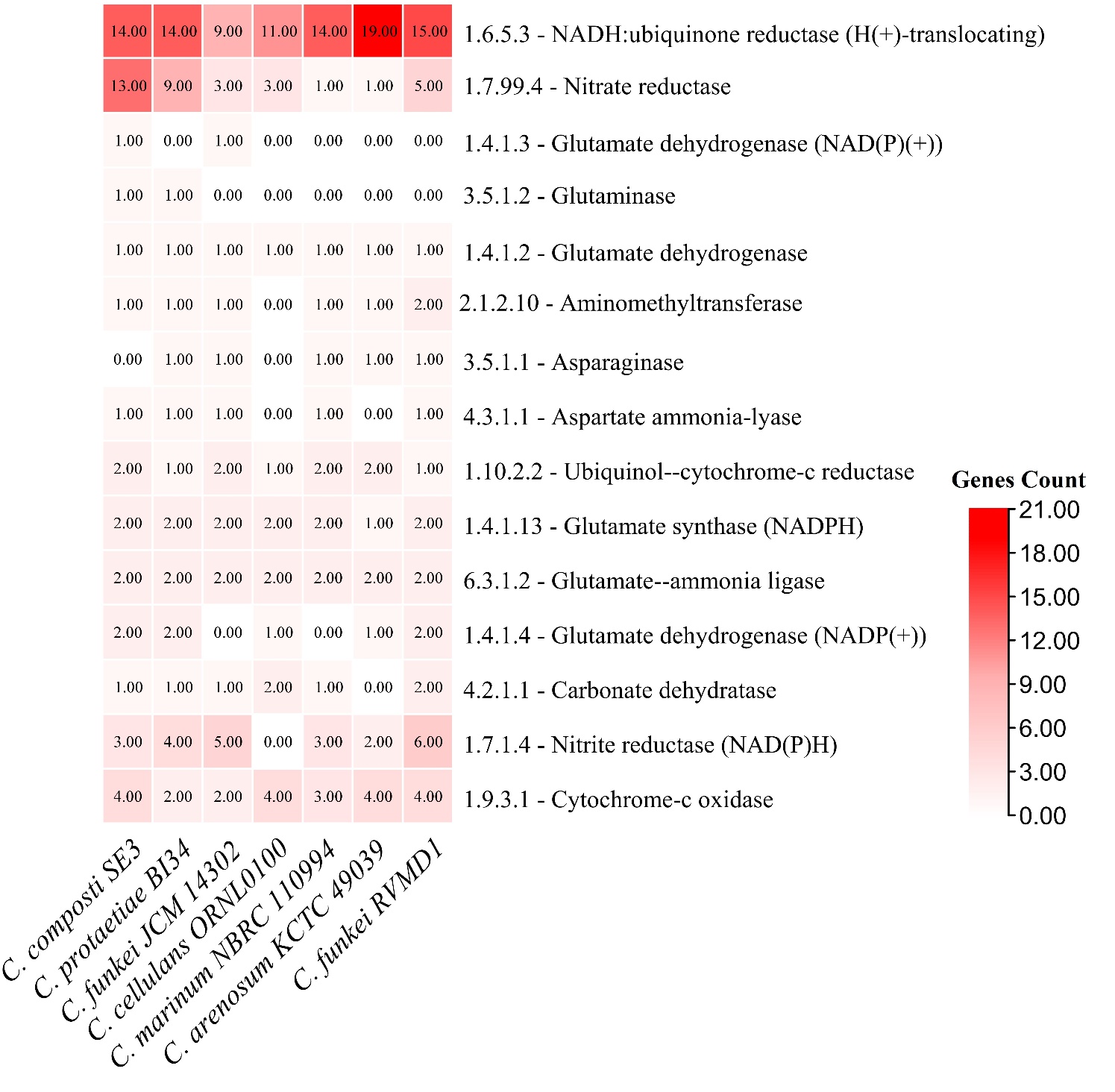

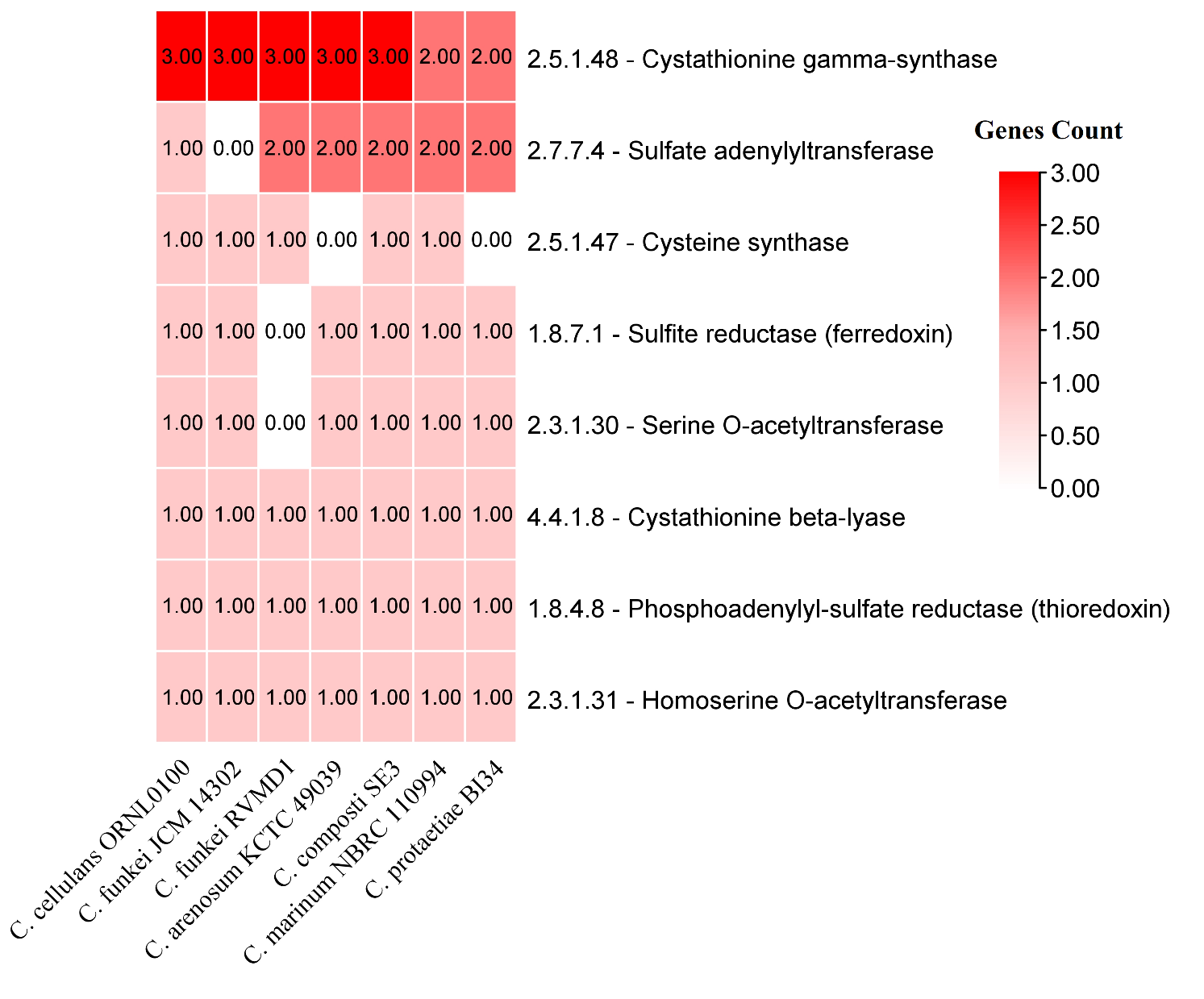
**

**A**

**B**

**Fig S8:** (A) Nitrogen metabolism gene comparison and (B) sulfur metabolism gene comparison in *C. funkei* RVMD1 against other reference strains across the *Cellulosimicrobium* genus. Nitrogen and sulfur metabolism comparisons were performed using the Pathway Comparison Tool in BV-BRC(Olson et al., 2023), and the results are represented as heatmap generated by TBtools-I(Chen et al., 2023).

**References**

Chen, C., Wu, Y., Li, J., Wang, X., Zeng, Z., Xu, J., et al. (2023). TBtools-II: A “one for all, all for one” bioinformatics platform for biological big-data mining. *J. Molecular. Plant.* 16(11)**,** 1733-1742. doi: 10.1016/j.molp.2023.09.010.

Ha, S.M., Kim, C.K., Roh, J., Byun, J.H., Yang, S.J., Choi, S.B., et al. (2019). Application of the Whole Genome-Based Bacterial Identification System, TrueBac ID, Using Clinical Isolates That Were Not Identified With Three Matrix-Assisted Laser Desorption/Ionization Time-of-Flight Mass Spectrometry (MALDI-TOF MS) Systems. *Ann. Lab. Med.* 39(6)**,** 530-536. doi: 10.3343/alm.2019.39.6.530.

Hitch, T.C.A., Riedel, T., Oren, A., Overmann, J., Lawley, T.D. and Clavel, T. (2021). Automated analysis of genomic sequences facilitates high-throughput and comprehensive description of bacteria. *ISME. Commun.* 20(1)**,** 16. doi: 10.1038/s43705-021-00017-z.

Medlar, A.J., Toronen, P. and Holm, L. (2018). AAI-profiler: fast proteome-wide exploratory analysis reveals taxonomic identity, misclassification and contamination. *Nucleic. Acids Res.* 46(W1)**,** W479-W485. doi: 10.1093/nar/gky359.

Olson, R.D., Assaf, R., Brettin, T., Conrad, N., Cucinell, C., Davis, J.J., et al. (2023). Introducing the Bacterial and Viral Bioinformatics Resource Center (BV-BRC): a resource combining PATRIC, IRD and ViPR. *Nucleic. Acids Res.* 51(D1)**,** D678-D689. doi: 10.1093/nar/gkac1003.

Parks, D.H., Imelfort, M., Skennerton, C.T., Hugenholtz, P. and Tyson, G.W. (2015). CheckM: assessing the quality of microbial genomes recovered from isolates, single cells, and metagenomes. *Genome Res.* 25(7)**,** 1043-1055. doi: 10.1101/gr.186072.114.

Rodriguez-R, L.M., Gunturu, S., Harvey, W.T., Rosselló-Mora, R., Tiedje, J.M., Cole, J.R., et al. (2018). The Microbial Genomes Atlas (MiGA) webserver: taxonomic and gene diversity analysis of Archaea and Bacteria at the whole genome level. *Nucleic. Acids Res.* 46(W1)**,** W282-W288.

Sun, J., Lu, F., Luo, Y., Bie, L., Xu, L. and Wang, Y. (2023). OrthoVenn3: an integrated platform for exploring and visualizing orthologous data across genomes. *Nucleic. Acids Res.* 51(W1)**,** W397-W403. doi: 10.1093/nar/gkad313.

Tamura, K., Stecher, G. and Kumar, S. (2021). MEGA11: Molecular Evolutionary Genetics Analysis Version 11. *Mol. Biol. Evol.* 38(7)**,** 3022-3027. doi: 10.1093/molbev/msab120.

Yoon, S.-H., Ha, S.-M., Kwon, S., Lim, J., Kim, Y., Seo, H., et al. (2017). Introducing EzBioCloud: a taxonomically united database of 16S rRNA gene sequences and whole-genome assemblies. *Int. J. Syst. Evol. Microbiol.* 67(5)**,** 1613.
